# The *Caenorhabditis elegans* Myc-Mondo/Mad Complexes Integrate Diverse Longevity Signals

**DOI:** 10.1371/journal.pgen.1004278

**Published:** 2014-04-03

**Authors:** David W. Johnson, Jesse R. Llop, Sara F. Farrell, Jie Yuan, Lindsay R. Stolzenburg, Andrew V. Samuelson

**Affiliations:** 1 University of Rochester, Department of Biomedical Genetics, Rochester, New York, United States of America; 2 Rochester Institute of Technology, Computer Science Department, Rochester, New York, United States of America; 3 Northwestern University, Feinberg School of Medicine, Chicago, Illinois, United States of America; University of California San Francisco, United States of America

## Abstract

The Myc family of transcription factors regulates a variety of biological processes, including the cell cycle, growth, proliferation, metabolism, and apoptosis. In *Caenorhabditis elegans,* the “Myc interaction network” consists of two opposing heterodimeric complexes with antagonistic functions in transcriptional control: the Myc-Mondo:Mlx transcriptional activation complex and the Mad:Max transcriptional repression complex. In *C. elegans,* Mondo, Mlx, Mad, and Max are encoded by *mml-1, mxl-2, mdl-1,* and *mxl-1,* respectively. Here we show a similar antagonistic role for the *C. elegans* Myc-Mondo and Mad complexes in longevity control. Loss of *mml-1* or *mxl-2* shortens *C. elegans* lifespan. In contrast, loss of *mdl-1* or *mxl-1* increases longevity, dependent upon MML-1:MXL-2. The MML-1:MXL-2 and MDL-1:MXL-1 complexes function in both the insulin signaling and dietary restriction pathways. Furthermore, decreased insulin-like/IGF-1 signaling (ILS) or conditions of dietary restriction increase the accumulation of MML-1, consistent with the notion that the Myc family members function as sensors of metabolic status. Additionally, we find that Myc family members are regulated by distinct mechanisms, which would allow for integrated control of gene expression from diverse signals of metabolic status. We compared putative target genes based on ChIP-sequencing data in the modENCODE project and found significant overlap in genomic DNA binding between the major effectors of ILS (DAF-16/FoxO), DR (PHA-4/FoxA), and Myc family (MDL-1/Mad/Mxd) at common target genes, which suggests that diverse signals of metabolic status converge on overlapping transcriptional programs that influence aging. Consistent with this, there is over-enrichment at these common targets for genes that function in lifespan, stress response, and carbohydrate metabolism. Additionally, we find that Myc family members are also involved in stress response and the maintenance of protein homeostasis. Collectively, these findings indicate that Myc family members integrate diverse signals of metabolic status, to coordinate overlapping metabolic and cytoprotective transcriptional programs that determine the progression of aging.

## Author Summary

Transcription factors are essential proteins that regulate the expression of genes and play an important role in most biological processes. The results of our study presented here demonstrate for the first time a role in aging for a small family of transcription factors in the nematode worm *Caenorhabditis elegans.* Importantly, these proteins have close relatives in higher organisms, including humans that influence metabolism, cell replication, and have been implicated in the development of cancer. Moreover, the loss of one homologue has also been implicated in Williams-Beuren syndrome, a disease characterized in part by signs of premature aging. Our data demonstrate that these transcription factors function within insulin/IGF-1 signaling and dietary restriction, two highly conserved pathways that link nutrient sensing to longevity. Taken together, our findings provide exciting new insight into a family of proteins that may be essential for linking nutrient sensing to longevity and have implications for the improvement of human healthspan.

## Introduction

A large body of evidence indicates that sensors and regulators of cell metabolism modulate organismal aging through evolutionarily conserved pathways. Research on the nematode *C. elegans* has been instrumental in the characterization of many of these pathways, including the insulin/IGF (ILS), Target of Rapamycin (TOR), AMP protein kinase (AMPK), sirtuin (Sir2), and dietary restriction (DR) pathways [1]–[5]. For example, the ILS and DR signaling pathways target the forkhead transcription factors DAF-16 (FoxO) and PHA-4 (FoxA), respectively, suggesting that these two regulatory inputs function independently [6]–[8]. Although much is known about the structure and function of these pathways, many questions remain about how those signals are integrated, the extent of their functional overlap, and how these pathways determine the progression of aging.

**Figure 1:**
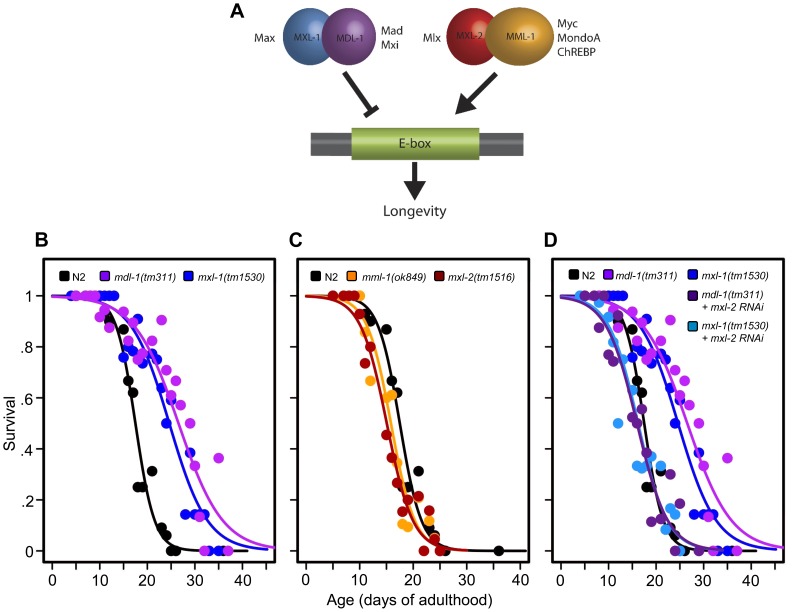
The *C. elegans* Myc-Mondo/Mad complexes have opposing functions in longevity. (A) The *C. elegans* Myc-Mondo/Mad interaction network consists of four proteins that form two heterodimeric complexes, with opposing effects on transcription and longevity. The Myc-Mondo transcriptional activation complex consists of MML-1 and MXL-2, and the Mad transcriptional repression complex consists of MDL-1 and MXL-1 [11]. The mammalian homologues are listed beside each protein. (B) The *mdl-1(tm311)* and *mxl-1(tm1530)* null mutations significantly increase lifespan (compare black (wild-type) to purple and blue, respectively). (C) The *mml-1(ok849)* and *mxl-2(tm1516)* null mutations significantly shorten lifespan (compare to wild-type (black)). (D) RNAi inactivation of *mxl-2* abolishes the lifespan extension conferred by *mdl-1(tm311)* and *mxl-1(tm1530)* null mutations (dark purple and dark blue, versus purple and blue traces respectively). Similar results were obtained using *mml-1* RNAi (Dataset S1). Graphs illustrate the combined data from multiple trials. Complete information regarding the number of trials, total number of animals examined, and statistical significance for each experiment can be found in Dataset S1.

The large Myc super-family is comprised of basic helix-loop-helix leucine zipper (bHLHZip) containing transcription factors that are important regulators of cell growth, proliferation, and energy metabolism. In mammals, there are over 100 bHLH transcription factors [9]. Within this larger super-family, the core mammalian “Myc interaction network” consists of 11 proteins: 3 Myc; 4 Mad/Mxi; 2 Mnt/Mga; and 2 Mondo proteins. These proteins can heterodimerize in a complex manner with either Max or Mlx to regulate transcription by binding to a range of enhancer box (E-box) DNA sequences. *C. elegans* possesses 42 bHLH transcription factors, including a simplified Myc interaction network [10] comprised of four genes: *mml-1* (T20B12.6, Myc and Mondo like), *mxl-2* (F40G9.11, Mlx), *mxl-1* (T19B10.11, Max), and *mdl-1* (R03E9.1, Mad) (Figure 1A). While *C. elegans* lacks a true Myc orthologue, *mml-1* contains a region that is highly similar to the N-terminal region of c-Myc, and presumably incorporates both Myc and Mondo functions [11]. Similar to their mammalian counterparts, members of the *C. elegans* Myc interaction network form obligate heterodimers that bind to a range of E-box sequences and either activate or repress transcription (respectively: MML-1:MXL-2, hereafter referred to as the Myc-Mondo complex and MDL-1:MXL-1, referred to as the Mad complex) (Figure 1A). Unlike mammalian Mlx and Max, these protein-protein interactions are specific, and no evidence exists for cross-heterodimerization. In *C. elegans,* null mutations in any of the four genes produces no overt developmental phenotype, although a subtle cell migration defect has been reported in *mxl-2(tm1516)* mutant animals [11]. From a comprehensive genome-wide RNAi screen, we had previously discovered that *mxl-2* is essential for the extended longevity of the *daf-2(e1370)* insulin/IGF1 receptor loss of function mutants [12]. Additionally, *mdl-1* has been putatively identified as a target of ILS based on microarray analysis, and loss of *mdl-1* has been reported to produce a subtle negative effect on the extension of lifespan in ILS mutants [13].

In mammals, two Mondo paralogs (MondoA and ChREBP) function as intracellular sensors of carbohydrate availability and regulators of glycolytic and lipogenic gene expression. MondoA (MLXIP) is predominately expressed in skeletal muscle. When activated by glucose-6-phosphate (G6P), it translocates from the mitochondrial outer membrane to the nucleus where it upregulates the expression of glycolytic genes, by DNA binding to E-box sequences. ChREBP (Carbohydrate Response Element Binding Protein/MLXIPL/MondoB/WBSCR14) is expressed in the liver and is also activated by carbohydrate intermediates, most likely G6P (reviewed in [14]). Activated ChREBP exhibits cytoplasmic-to-nuclear shuttling similar to MondoA and upregulates the expression of both glycolytic and lipogenic genes (triglyceride synthesis) through binding of carbohydrate response elements (ChORE sequences) within the genome [15]–[17]. In mammals, the absence of ChREBP ablates glucose-induced target gene activation [18], [19]. ChREBP –/– mice display marked physiological changes: large glycogen-laden livers; smaller fat deposits; decreased plasma free fatty acid levels and lipogenic enzyme expression; signs of insulin resistance; reduced glycolytic flux; and enhanced glycogen accumulation [20]. While the specific roles of ChREBP in humans have not been elucidated, the gene encoding ChREBP is one of 27 genes deleted in Williams-Beuren Syndrome (WBS), a complex developmental disorder. Interestingly, WBS patients present signs of premature aging including: premature graying of hair; glucose intolerance; diabetes; and hearing loss that commonly develops during adolescence or young adulthood, concomitant with declining memory skills or dementia [21].

## Results

### *C. elegans* Myc-Mondo and Mad Complexes have Opposing Roles in Longevity

Prompted by our previous identification of *mxl-2* as an effector of *daf-2* mutants in the extension of *C. elegans* lifespan [12], we asked whether Myc-Mondo and Mad complexes might affect longevity. Thus we obtained null mutant animals for *mml-1*, *mxl-2*, *mdl-1*, and *mxl-1*, then assessed their lifespan. Loss of either component of the transcriptional repressor complex resulted in a robust extension of lifespan: *mdl-1(tm311)* and *mxl-1(tm1530)* single null mutant animals had a 44.5% and 33.2% (p<0.0001) increase in median lifespan compared to wild-type (N2) animals, which had a median lifespan of 18.4+/–0.4 days (Figure 1B, Dataset S1). Using traditional and replica set lifespan approaches, similar results were obtained when either *mdl-1* or *mxl-1* were inactivated by RNAi (Figure S6B, S6C, Dataset S1). Furthermore, simultaneous inactivation of *mxl-1* and *mdl-1* resulted in lifespans comparable to the single mutant animals (Figure S1A, S1B, Dataset S1), consistent with published observations in *C. elegans* that these two proteins only function as an obligate heterodimer [10], [11]. In contrast, loss of either component of the transcriptional activation complex shortened lifespan; *mml-1(ok849)* and *mxl-2(tm1516)* single null mutant animals had a 14.1% and 22.8% reduction in median lifespan compared to wild-type animals (Figure 1C, Dataset S1). Inactivation of *mml-1* or *mxl-2* by RNAi produced a similar result using both traditional and replica set lifespan analyses (Figure S6, Dataset S1). Analogous to what was observed with *mdl-1* and *mxl-1*, simultaneous inactivation of *mml-1* and *mxl-2* resulted in lifespans comparable to the single mutant animals (Figure S1C, S1D, Dataset S1). Thus loss of the Mad (MDL-1:MXL-1) transcriptional repressor complex extends lifespan, while loss of the Myc-Mondo (MML-1:MXL-2) activator complex shortens lifespan.

We conducted an epistasis analysis to determine whether the lifespan extension conferred by loss of the Mad complex (MDL-1:MXL-1) was dependent on the function of the Myc-Mondo complex (MML-1:MXL-2). Inactivation of either *mml-1* or *mxl-2* via RNAi fully suppressed the extended longevity of both *mdl-1(tm311)* and *mxl-1(tm1530)* mutant animals (Figure 1D, Dataset S1). Therefore, the extended longevity conferred by the loss of the Mad transcriptional repression complex is dependent upon an intact Myc-Mondo complex. Interestingly, *C. elegans* possess a second Max like protein, MXL-3 (F46G10.6) [22]. Both MXL-2 and MXL-3 share similar sequence homology to mammalian Max; however, MXL-3 likely functions as a homodimer unlike MXL-2 and mammalian Max [10]. Moreover, *mxl-3* links lipolysis and autophagy to nutrient availability [10], [23], and *mxl-3(ok1947)* null mutant animals are also long-lived (Figures S1E, S1F, Dataset S1 and [23]). In contrast to *mdl-1* or *mxl-1* mutants, inactivation of either *mml-1* or *mxl-2* had a negligible effect on *mxl-3(ok1947)* longevity (Figures S1E, S1F, Dataset S1). Thus the Myc-Mondo transactivation complex is required for the extension of lifespan in the absence of the Mad complex. In contrast, Myc-Mondo complex function is dispensable for the extension of lifespan conferred by loss of the Max paralog *mxl-3.* Collectively, this implies that the Myc-Mondo and Mad complexes have similar functions that influence longevity, which are separate from those of the Max paralog *mxl-3.*

### The Myc-Mondo and Mad Complexes Functionally Overlap with ILS-Mediated Longevity

**Figure 2:**
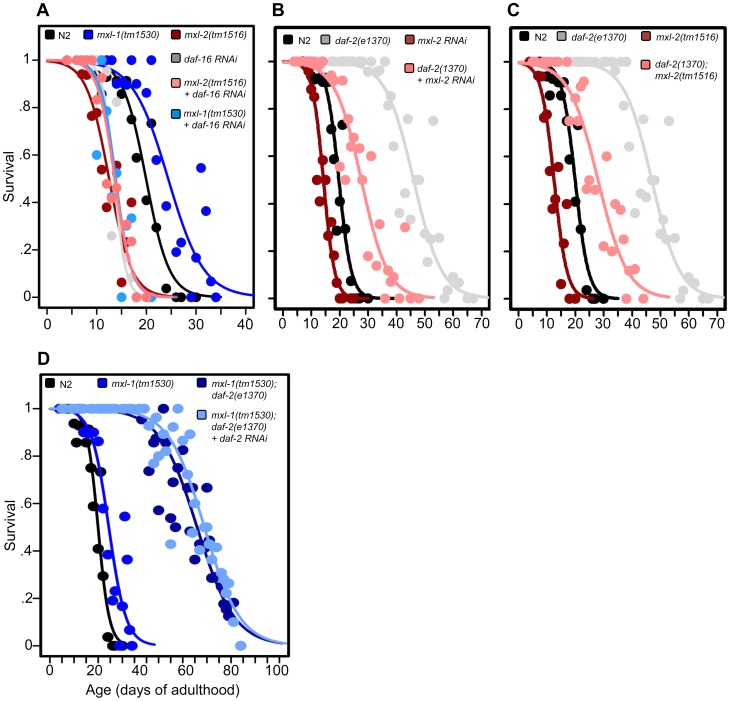
The Myc-Mondo/Mad complexes intersect with ILS in longevity. (A) RNAi inactivation of *daf-16* ablates the lifespan extension conferred by *mxl-1(tm1530)* null mutation (dark blue versus blue traces), and does not further shorten the lifespan of *mxl-2(tm1516)* null mutants (pink versus red traces). Reciprocal experiments show that RNAi inactivation of *mxl-1* or *mdl-1* do not extend the lifespan of *daf-16(mgDf47)* null mutant animals, nor does RNAi inactivation of *mxl-2* or *mml-1* further shorten the lifespan of *daf-16(mgDf47)* null mutant animals (Dataset S1). (B) RNAi inactivation of *mxl-2* partially suppresses the lifespan of *daf-2(e1370)* mutant animals (pink versus gray traces). Similar results were obtained with *mml-1* RNAi (Figure S2A). (C) The *mxl-2(tm1516)* null mutation partially suppresses the extended lifespan observed in *daf-2(e1370)* mutant animals (pink versus gray traces). (D) RNAi inactivation of *daf-2* does not further extend the lifespan of *mxl-1(tm1530); daf-2(e1370)* mutant animals (darker blue versus light blue traces). Traces represent the combined data from multiple separate trials. Complete information regarding the number of trials, total number of animals examined, and statistical significance for each experiment can be found in Dataset S1. Experiments shown within this figure were performed simultaneously with those shown in Figure 3 and were split into multiple figures for readability.

We next determined whether the Myc-Mondo and Mad complexes functioned in insulin/IGF1 signaling, which is known to depend on the FoxO homologue *daf-16*. First we asked whether *daf-16* was necessary for loss of the Mad complex (MDL-1:MXL-1) to extend longevity; second, we asked whether inactivation of *daf-16* further shortened longevity in the absence of the Myc-Mondo complex (MML-1:MXL-2). Inactivation of *daf-16* by RNAi completely suppressed the extended lifespan of *mxl-1(tm1530)* mutant animals (Figure 2A, Dataset S1). Similarly, inactivation of either *mdl-1* or *mxl-1* failed to confer longevity in *daf-16(mgDf47)* null mutant animals (Dataset S1). Conversely, *daf-16(RNAi)* did not further shorten the lifespan of *mxl-2(tm1516)* null mutant animals (Figure 2A, Dataset S1) and neither *mml-1(RNAi)* nor *mxl-2(RNAi)* further shortened the longevity of *daf-16(mgDf47)* null mutant animals (Dataset S1). Thus *daf-16* (FoxO) is essential for loss of the Mad complex to extend longevity, and loss of the Myc-Mondo complex has no further negative effect on longevity in the absence of *daf-16*.

To further study the role of the Myc proteins in the longevity effects of ILS, we asked whether the transcription-activating Myc-Mondo complex (MML-1:MXL-2) was required for *daf-2(e1370)* extension of lifespan. *daf-2(e1370)* mutant animals fed control RNAi had a median and maximum lifespan of 46.0 +/– 1.2 days and 60.5 +/–2.4 at 20°C, respectively (Figure 2B, Dataset S1). Inactivation of either *mxl-2* or *mml-1* by RNAi reduced the median lifespan of *daf-2(e1370)* mutant animals, to 27.6+/–0.9 days and 27.2+/–1.3 days, respectively; a result consistent with our previous findings (Figures 2B, S2A, Dataset S1, and [12]). Similarly, *daf-2(e1370);mxl-2(tm1516)* double mutant animals had a median lifespan of 28.0+/–1.4 days (Figure 2C, Dataset S1). Thus loss of Myc-Mondo complex function by null mutation reduces *daf-2(e1370)* median lifespan by 18.0+/–1.4 days and wild-type lifespan by 4.2+/–0.5 days. The reduction caused by loss of *mxl-2* is significantly greater (p = 0.001) in the *daf-2(e1370)* background (39.1%) than in the wild-type background (22.8%). This implies that the effect of the loss of the Myc-Mondo complex on lifespan has specificity to insulin/IGF-1 signaling. This is consistent with the results of a global analysis of synthetic genetic interactions [24], which predicted a relationship between *mml-1* and *daf-2*.

If, as suggested by the above experiments, *daf-2* and the Myc proteins are part of a common lifespan regulating system, they would not be expected to act independently. We sought to confirm this hypothesis by testing whether there is additivity between loss of the Mad complex and decreased ILS signaling. However, interpreting an epistasis analysis with ILS mutants is complicated, as even strong temperature-sensitive (ts) alleles of *daf-2* [25] retain some level of ILS. For example, placing *daf-2(e1370)* mutant animals onto *daf-2(RNAi)* results in a 21.8% increase in median longevity from 46.0+/–1.2 to 56.0+/–1.6 days at 20°C (p = 0.0032) (Figure S2B). In order to perform *daf-2* pathway analysis with the lowest sub-lethal amount of insulin-like signaling we examined the lifespan of *daf-2(e1370)* and *daf-2(e1370);mxl-1(tm1530)* mutants in the presence of *daf-2* RNAi. Unlike *daf-2(e1370)* single mutant animals, *daf-2(e1370);mxl-1(tm1530)* double mutant animals show no significant further increase in lifespan on *daf-2(RNAi)* (p = 0.1164) (Figure 2D), implying that the *C. elegans* Mad complex and decreased ILS have similar functions in longevity.

### The Myc-Mondo and Mad Complexes Contribute to DR-Mediated Longevity

**Figure 3:**
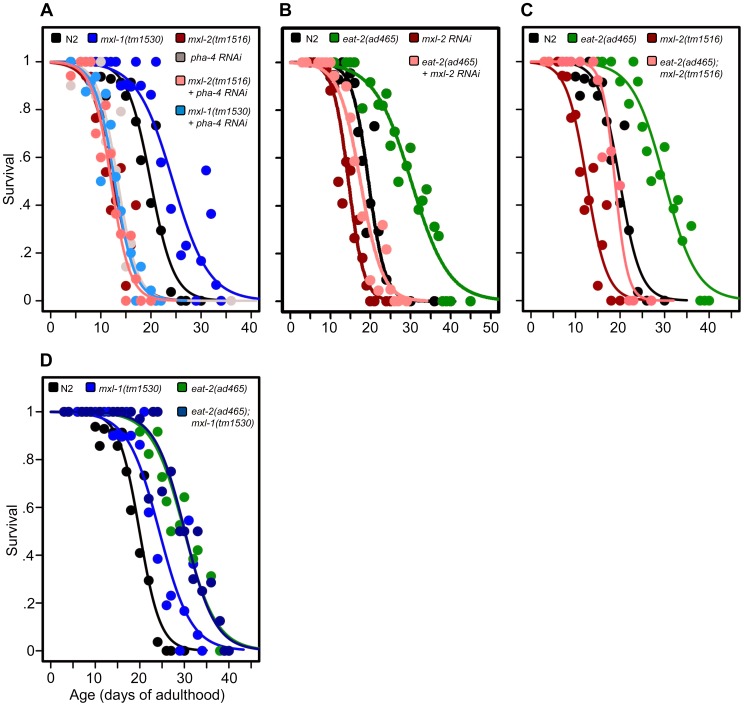
The Myc-Mondo/Mad complexes intersect with dietary restriction in longevity. (A) RNAi inactivation of *pha-4* ablates the lifespan extension conferred by the *mxl-1(tm1530)* null mutation (light blue versus blue traces), and does not further shorten the lifespan of *mxl-2(tm1516)* null mutant animals (pink versus red traces). (B) RNAi inactivation of *mxl-2* partially suppresses the extended lifespan observed in *eat-2(ad465)* mutants (pink versus green traces). Similar results were obtained with *mml-1* RNAi (Dataset S1). (C) The *mxl-2(tm1516)* null mutation partially suppresses the extended lifespan observed in *eat-2(ad465)* mutant animals (pink versus green traces). (D) The *mxl-1(tm1530)* null mutation does not further extend the lifespan of *eat-2(ad465)* mutant animals (dark blue versus green traces). Similar results were obtained with *mxl-1* and *mdl-1* RNAi (Dataset S1). Traces represent the combined data from multiple separate trials. Complete information regarding the number of trials, total number of animals examined, and statistical significance for each experiment can be found in Dataset S1. Experiments shown within this figure were performed simultaneously with those shown in Figure 2 and were split into multiple figures for readability.

As mammalian MondoA and ChREBP are known sensors of carbohydrate availability [14], we hypothesized that the Myc-Mondo (MML-1:MXL-2) and Mad (MDL-1:MXL-1) complexes may function in DR signaling. The *pha-4* (FoxA) transcription factor is a major transcriptional output of DR signaling [8]. Analogous to our analysis with *daf-16(RNAi)* and ILS, we conducted epistasis experiments with *pha-4(RNAi)* to determine whether the Myc-Mondo and Mad complexes function in DR signaling. Inactivation of *pha-4* completely suppressed the extended lifespan of both *mxl-1(tm1530)* (p = 0.0001) and *mdl-1(tm311)* (p = 0.0002) mutant animals, similar to our results with *daf-16(RNAi)* (Figure 3A, Dataset S1). Additionally, *pha-4(RNAi)* did not further shorten the lifespan of *mxl-2(tm1516)* mutant animals (p = 0.5835) (Figure 3A, Dataset S1). Thus *pha-4* is required for the extension of lifespan conferred by loss of the Mad complex, and has similar pro-longevity functions as the Myc-Mondo complex.

Next, we tested whether the Myc-Mondo (MML-1:MXL-2) complex is required for lifespan extension by DR. To do this, we conducted a lifespan analysis in *eat-2(ad465)* mutant animals, which harbor a mutation in a nicotinic acetylcholine receptor, resulting in drastically decreased pharyngeal pumping and increased lifespan [26], [27]. Notably, *eat-2(ad465);mxl-2(tm1516)* and *eat-2(ad465);mxl-1(tm1530)* mutant animals have pumping rates identical to those seen in *eat-2(ad465)* mutants (Figure S7). Loss of the Myc-Mondo complex by *mxl-2(RNAi)*, or *eat-2(ad465);mxl-2(tm1516)* double mutant partially suppressed the DR-mediated lifespan extension (Figure 3B, 3C, Dataset S1) by 11.8+/–1.3 days (40.5%), and 10.3+/–1.3 days (35.4%), respectively. This reduction is significantly greater than the 4.1+/–0.5 day decrease (22.7% reduction) in lifespan that results from the loss of *mxl-2* in an otherwise wild-type background (p = 0.00055). Thus the Myc-Mondo complex is required for the increased lifespan conferred by DR, similar to what we found with ILS.

Lastly, we tested whether there would be additivity to overall lifespan in the absence of the Mad complex (MDL-1:MXL-1) under conditions of DR. Inactivation of either *mxl-1* or *mdl-1* did not significantly extend *eat-2(ad465)* lifespan, with median lifespans of 29.1+/–1.1; 29.3+/–1.1 (p = 0.398); 32.0+/–2.2 days (p = 0.28), respectively (Dataset S1). Additionally, *eat-2(ad465)* and *eat-2(ad465);mxl-1(tm1530)* mutant animals have similar lifespans (median lifespans of 29.1+/–1.1, and 30.1+/–1.3 (p = 0.565), Figure 3D, Dataset S1). Collectively, these results indicate that, in addition to ILS, *C. elegans* Myc-Mondo and Mad complexes function in DR longevity signaling.

### TGF-β Signaling Does Not Influence Lifespan by Suppressing Mad Complex Function

The TGF-β pathway functions in parallel with ILS to regulate energy-balance, thereby affecting dauer formation, fat metabolism, egg laying, feeding behavior, and lifespan [13],[28]–[31]. *daf-7* encodes a member of the TGF-β superfamily, which inactivates the co-SMAD DAF-3 via the TGF-β Type I and II receptors DAF-1 and DAF-4. Loss of *daf-7* extends longevity dependent upon *daf-3* [32]. Interestingly, DAF-3 binds the *mdl-1* promoter, and *mdl-1* expression in the pharynx is increased in *daf-3* RNAi-treated animals, suggesting that DAF-3 negatively regulates the expression of *mdl-1* [33]. Thus the decreased longevity of *daf-3(tm4940)* null mutant animals might, at least in part, be explained by upregulation of the Mad complex, and we predicted that loss of the Mad complex might rescue the shortened longevity of *daf-3(tm4940)* animals. To test this hypothesis, wild-type and *daf-3(tm4940)* null mutant animals were treated with control and *mxl-1* RNAi, and lifespan was assessed. *mxl-1* RNAi robustly increased the lifespan of wild-type animals by 5.0+/–0.9 days (27.3% increase, p<0.0001, Figure S3, Dataset S1), which is comparable to the effect of the *mxl-1* null mutation (Figure 1B, Dataset S1). In contrast, inactivation of *mxl-1* failed to extend the lifespan of *daf-3(tm4940)* null mutant animals (Figure S3, p = 0.24). Therefore, we conclude that TGF-β signaling does not influence lifespan through regulation of the Mad complex.

### Decreased ILS or Conditions of DR Promote Nuclear Accumulation of MML-1 (Myc-Mondo), Dependent on *pha-4*

**Figure 4:**
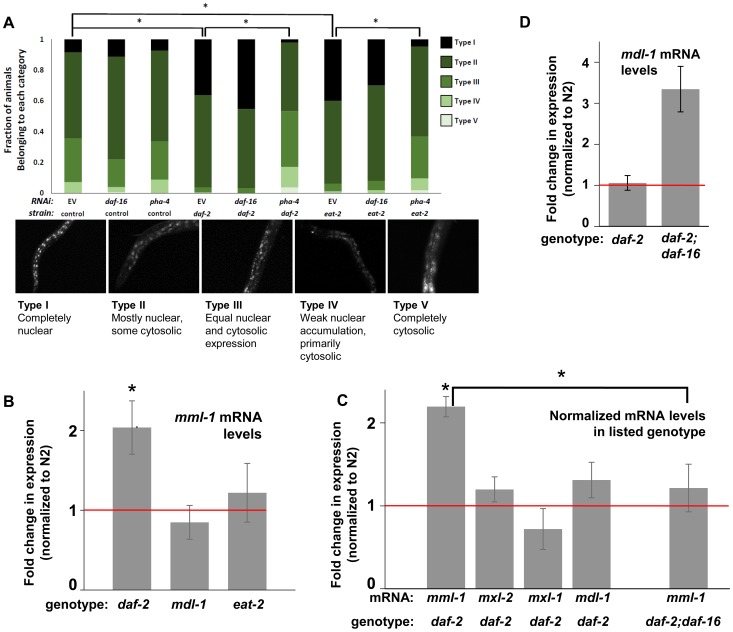
ILS and DR pathways differentially regulate *mml-1* gene expression and MML-1 localization. (A) Nuclear accumulation of MML-1::GFP is increased in *daf-2(e1370)* and *eat-2(ad465)* mutant animals. Loss of *pha-4*, but not *daf-16*, blocks increases in MML-1::GFP nuclear accumulation in *daf-2(e1370)* and *eat-2(ad465)*, but does not affect MML-1::GFP localization under basal conditions. Representative images of Types I-V are found below the graph. Additional high-resolution images of MML-1::GFP expressing animals can be found in File S1 (* denotes p < 0.05). (B) *mml-1* mRNA levels are significantly increased in *daf-2(e1370)*, but not in *mdl-1(tm311)* or *eat-2(ad465)* mutant animals, as compared to N2 (* denote p<0.05). (C) *mxl-2, mxl-1,* and *mdl-1* mRNA levels are unaffected in *daf-2(e1370)* mutants. A *daf-16* null mutation suppresses the increases in *mml-1* mRNA observed in *daf-2(e1370)* mutants. (* denotes p<0.01) (D) *mdl-1* mRNA levels are significantly increased in *daf-2(e1370);daf-16(mgDf47)* mutants (* denotes p<0.01).

In mammals, the MondoA/ChREBP:Mlx complexes exhibit translocation from cytoplasm to nucleus in response to changes in carbohydrate availability [34], [35]. We hypothesized that changes in metabolic status, as relayed through decreased ILS or conditions of DR, might alter MML-1 subcellular localization. To determine whether the *C. elegans* Myc-Mondo complex (MML-1:MXL-2) might be regulated by a similar translocation, we generated an MML-1::GFP translational reporter under control of the endogenous *mml-1* gene promoter. As has been previously reported, MML-1::GFP is broadly expressed (Figure 4A and [11]). However, contrary to previous reports, MML-1::GFP was observed to reside in both the nucleus and the cytoplasm under basal conditions (Figure 4A). The MML-1::GFP fusion was functional, as it rescued the thermotolerance defect of *mml-1(ok849)* (data not shown). In control animal populations, the relative distribution of MML-1::GFP varied between animals, ranging from entirely nuclear to entirely cytoplasmic; however, subpopulations showed predominately either nuclear with some cytoplasmic (56.1%), or approximately equal distribution between the nucleus and cytoplasm (28.3%) (Figure 4A, File S1). In contrast, decreased ILS (*daf-2(e1370)*) or conditions of DR (*eat-2(ad465)*) significantly increased the prevalence of the subpopulation with entirely nuclear MML-1::GFP from 8.3% to 36.1% and 40.0%, respectively (Figure 4A, compare columns 1 to 4 and 1 to 7). The increased nuclear accumulation of MML-1::GFP in response to reduced ILS and conditions of DR supports the idea that this aspect of MML-1/MondoA/ChREBP biology is conserved in nematodes, and provides a potential mechanistic explanation for the requirement of the Myc-Mondo complex in ILS and DR-mediated longevity; specifically that Myc-Mondo complex function is activated by decreased ILS or by conditions of DR to increase the expression of genes that extend longevity.

We hypothesized that the nuclear translocation of MML-1 in conditions of decreased ILS might be dependent upon *daf-16,* while increased nuclear accumulation in conditions of DR would require *pha-4.* Inactivation of either *daf-16* or *pha-4* failed to significantly alter the basal spectrum of MML-1::GFP nuclear accumulation in control animals (Figure 4A compare columns 2 and 3 to 1). To our surprise, inactivation of *daf-16* failed to alter the increased nuclear accumulation of MML-1::GFP conferred by decreased ILS or conditions of DR (Figure 4A compare column 4 to 5 and column 7 to 8). In contrast, inactivation of *pha-4* completely abolished the increased nuclear accumulation of MML-1::GFP conferred by decreased ILS or conditions of DR back to the distributions found in control populations (Figure 4A, compare column 4 to 6 and column 7 to 9). Thus we conclude that *pha-4,* but not *daf-16,* is essential for the increased nuclear accumulation of MML-1 under conditions of DR or decreased ILS.

### DAF-16 Activity Regulates the Expression of Myc Family Members

We next asked whether ILS or DR influenced the expression of Myc family members. Interestingly, according to the modENCODE database (www.modENCODE.org) only the *mml-1* promoter is directly bound by DAF-16, PHA-4, and MDL-1, making it the most likely target for transcriptional regulation in response to aging-relevant signals, such as ILS and DR [36]. Thus we first assessed whether decreased ILS, DR, or loss of *mdl-1* altered expression of *mml-1* by qRT-PCR. In *daf-2(e1370)* mutant animals, *mml-1* mRNA levels were significantly increased by 2-fold (Figure 4B, p = 0.0027). In contrast, a significant change in *mml-1* mRNA levels was not detected in *mdl-1(tm311),* or *eat-2(ad465),* mutant animals (Figure 4B, p = 0.3817 and 0.6033). To test whether the increased mRNA expression under conditions of decreased ILS was unique to *mml-1*, we also used qRT-PCR to measure the relative mRNA levels of *mxl-2*, *mxl-1*, and *mdl-1* in *daf-2(e1370)* compared to wild-type animals. Only *mml-1* was significantly upregulated under conditions of low ILS (Figure 4C, p = 0.0024); this implies that insulin-like signaling only transcriptionally regulates Myc-Mondo complex function at the level of *mml-1* expression. Consistent with that notion, *mxl-2* and *mxl-1* are not direct targets of DAF-16 as measured by ChIP-seq (www.modENCODE.org), although *mxl-1* is the last gene in an operon that is a target of DAF-16. Therefore, it is possible that ILS regulates *mxl-1* expression within the context of the operon at a level that cannot be distinguished by qRT-PCR.

Since decreased ILS, but not conditions of DR, regulated the Myc family at the mRNA level, we explored the role of DAF-16 in this regulation. We first determined in qRT-PCR experiments whether the upregulation of *mml-1* by decreased ILS was *daf-16* dependent. The increased *mml-1* mRNA levels observed in *daf-2(e1370)* mutant animals is *daf-16* dependent, as *daf-2(e1370);daf-16(mgDf47)* double mutant animals expressed *mml-1* at levels comparable to wild-type control animals (Figure 4C). Thus we conclude that decreased ILS activates DAF-16 to induce expression of *mml-1*, perhaps as a feed-forward mechanism to amplify DAF-16 signaling. Since MML-1 (Mondo) and MDL-1 (Mad) have opposing roles in transcription and longevity control, we hypothesized that loss of *daf-16* might induce expression of *mdl-1.* Indeed, *daf-2(e1370);daf-16(mgDf47)* double mutant animals have a 3-fold increase in *mdl-1* mRNA levels (Figure 4D, p = 0.0013). DAF-16 binds in the promoter region of *mdl-1* (http://www.modENCODE.org and [37]). Thus we conclude that DAF-16 activity regulates expression of the key components of the Myc-Mondo and Mad complexes in a manner that correlates with their role in longevity. Specifically, decreased ILS activates DAF-16, which in turn results in increased *mml-1* expression and extension of longevity; in contrast, loss of *daf-16* results in increased *mdl-1* expression and shortened lifespan.

### DAF-16, PHA-4, and MDL-1 Genomic Binding Overlaps at Metabolic and Cytoprotective Target Genes

**Figure 5:**
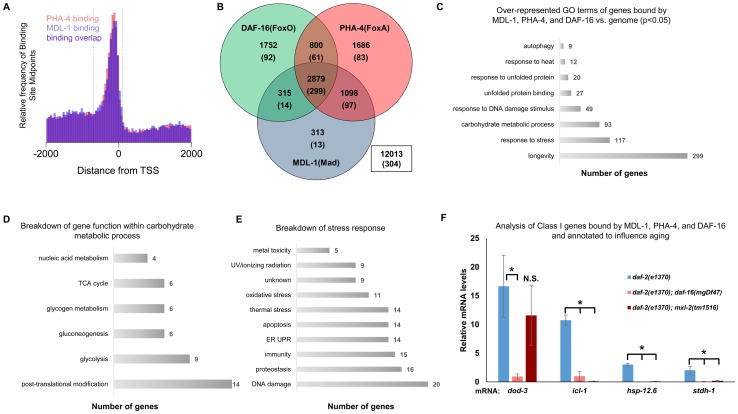
ChIP-seq data shows significant overlap amongst MDL-1, DAF-16, and PHA-4 promoter binding. (A) Analysis of binding sites throughout the genome indicates that MDL-1 (blue) and PHA-4 (red) bind predominantly in a region located –700 to +100 bp (dashed lines) relative to the transcriptional start site (TSS). (B) Venn diagram based on ChIP-Seq data showing gene promoters bound by MDL-1, DAF-16, and PHA-4. Numbers indicate the total number of genes belonging to each group. Parenthesis denote the number of genes belonging to each group that have previously been annotated to be involved in aging (based on published studies, gene ontology and description). The inset box shows the total number of genes in the genome that are not bound by MDL-1, DAF-16, and PHA-4. (C) GO terms related to aging that are enriched in genes bound by MDL-1, DAF-16, and PHA-4 versus the entire genome (p <0.05). The total number of common target genes annotated with each GO term is shown to the right of each bar. Enrichment was determined as described in Materials and Methods. Details of this analysis can be found in Dataset S2 (D) Analysis of metabolic pathways contained within the GO term “carbohydrate metabolic process” (GO: 0005975). The total number of genes annotated within each pathway is shown. Some genes belong to more than one category, notably several genes function in glycolysis and gluconeogenesis. Details can be found in Dataset S2 (E) An analysis of stress response pathways contained with the GO term “response to stress” (GO: 0006950). The total number of genes annotated to be involved in each pathway is shown. Many genes are important for resistance to multiple stresses. Details can be found in Dataset S2. (F) qRT-PCR analysis of Class I genes [37] known to be bound by MDL-1, DAF-16, and PHA-4 in *daf-2(e1370), daf-2(e1370);daf-16(mgDf47),* and *daf-2(e1370);mxl-2(tm1516)* animals. Class I genes were significantly upregulated (* denotes p<0.01) in *daf-2(e1370)* in a *daf-16* dependent manner as previously described [13], [37]. Additionally, *icl-1, hsp-12.6, and stdh-1* failed to be induced in *daf-2(e1370);mxl-2(tm1516)* mutants as well.

We found that the Myc family members are regulated by DR and ILS signaling and have overlapping functions in longevity control, implying that the *C. elegans* Myc-Mondo/Mad complexes may cooperate with PHA-4 and DAF-16 at shared target gene promoters. Through an informatics analysis of available ChIP-seq data provided by the modENCODE project (www.modENCODE.org) [38], we sought to identify the overlap in genomic binding sites for DAF-16, PHA-4, and MDL-1. We compared the global frequency of the MDL-1 and PHA-4 binding midpoints to the position of the transcription start site (TSS), as has been done for DAF-16 [37]. MDL-1 and PHA-4 had enriched binding frequency within a region almost identical to what has been reported for DAF-16 (between –700 to +100 of the TSS, Figure 5A). We used this metric to distinguish genes more likely to be regulated by MDL-1, and identified 4605 possible target genes. The Myc family of bHLH transcription factors bind to E box sequences, therefore we asked whether the MDL-1 binding regions identified by ChIP-seq also contained E boxes, and found this to be true in 3555 cases (77.2%, Dataset S2). Next, we found that there is significant overlap of 2879 target genes that are bound by DAF-16, PHA-4, and MDL-1 (Figure 5B, p = 2.2e^−16^). Of 963 genes that have been identified as influencing longevity (based on published studies, gene ontology and description), 299 are bound by DAF-16, PHA-4, and MDL-1. In contrast, only *13* previously identified longevity genes are bound by MDL-1 and not DAF-16 or PHA-4, a significant enrichment (p = 2.2e^−16^). We next asked whether the actual binding sites of the three transcription factors overlapped. For 234 of 299 putative longevity genes the MDL-1 binding peak is 75% or more overlapped by DAF-16 and PHA-4, suggesting that binding occurs in common regulatory regions. We conclude that DAF-16, PHA-4, and MDL-1 bind at many common target genes, in common regions.

To gain mechanistic insight into the potential transcriptional programs co-regulated by DAF-16, PHA-4, and MDL-1, we determined whether there was over-representation of aging related gene ontology terms at putative common target genes. Comparing gene ontology of genes bound by DAF-16, PHA-4, and MDL-1 versus all genes revealed that determination of adult lifespan, carbohydrate metabolism, autophagy, unfolded protein binding, and stress response were significantly over-represented (Figure 5C and Dataset S2). An analysis of all potential MDL-1 targets (an additional 1726 genes) revealed similar classes of over-represented gene functions. Further, comparing MDL-1 target genes to previously identified putative targets of mammalian Mad1 [39], [40] reveals a large degree of overlap, which implies evolutionary conservation (Dataset S2). This suggests that DAF-16, PHA-4, and the Myc family converge on a significant subset of common target genes that have functions relevant to aging, carbohydrate metabolism, proteostasis, and stress response.

An examination of the specific carbohydrate metabolic pathways putatively regulated by MDL-1, DAF-16, and PHA-4 revealed a number of genes involved in glycolysis, gluconeogenesis, and glycogen metabolism (Figure 5D, Dataset S2). This is consistent with the reported function of the mammalian Mondo/ChREBP complexes, which are essential for the expression of genes in the same metabolic pathways [18]–[20], [41], [42]. Additionally, our analysis identified a number of stress response pathways that may be influenced by MDL-1 in conjunction with DAF-16 and PHA-4, including DNA damage response, proteostasis, unfolded protein response (UPR), oxidative stress, and thermotolerance (Figure 5E, Dataset S2).

If DAF-16 and Myc family members converge on common target genes, then they should have similar functions in longevity control. We tested this hypothesis by assessing whether overexpression of a DAF-16::GFP translational fusion could rescue the shortened lifespan that occurs in the absence of the Myc-Mondo activation complex. Compared to wild-type control, *daf-16* overexpression increased lifespan by 6 days (median lifespan of 17 and 23 days, respectively a 35.3% increase, p<0.0001) consistent with published findings [43]. Interestingly, overexpression of *daf-16* completely rescued the shortened lifespan of the *mxl-2(tm1516)* null mutation (Figure S4). Additionally, the *mxl-1(tm1530))* null mutation did not further extend lifespan when *daf-16* was over-expressed (Figure S4). Thus over-expression of *daf-16* is sufficient to compensate for the lack of Myc-Mondo transactivation, and loss of the Mad transcriptional repression complex does not further extend lifespan when *daf-16* is overexpressed, which is consistent with the notion that these transcription factors may act at common target genes.

We next asked whether Myc-Mondo function was necessary for the activation of DAF-16 target genes under conditions of decreased ILS. To this end, we measured mRNA expression levels of several candidates by qRT-PCR, which were chosen based on the criteria that they:are bound by DAF-16, PHA-4, and MDL-1 (modENCODE and [37]); are upregulated under conditions of decreased ILS in a *daf-16* dependent manner (i.e. are “*Class 1*” DAF-16 target genes, [37]); and have been implicated in the determination of adult longevity (GO term: 0008340). Based on these criteria five candidate genes were chosen: *dod-3,* an apparently nematode-specific gene previously shown to be “downstream of *daf-16*” [13]; *icl-1*, encoding isocitrate lyase and malate synthetase, which together form the glyoxylate shunt, an alternative metabolic pathway known to be favored in *daf-2* mutants, dietarily restricted worms, and Mit mutants [13], [44], [45]; *sodh-1* which encodes a sorbitol dehydrogenase; *hsp-12.6*, a small heat-shock protein; and *stdh-1*, a sterol dehydrogenase. Consistent with published findings, all but *sodh-1* were induced by decreased ILS in a *daf-16* dependent manner [37]. Of these four genes, the expression of three (*icl-1, hsp-12.6,* and *stdh-1*) were also suppressed in *daf-2(e1370);mxl-2(tm1516)* double mutant animals (Figure 5F). Additionally, we discovered that Myc-Mondo complex was not necessary for the increased nuclear accumulation of DAF-16 under conditions of decreased ILS, as GFP was solely nuclear in *daf-2(e1370);DAF-16::GFP* and *daf-2(e1370);mxl-2(tm1516);DAF-16::GFP* mutant animals (data not shown). We conclude that the Myc-Mondo transcriptional activation complex is necessary for the activation of at least a subset of DAF-16 target genes under conditions of decreased ILS.

### The *C. elegans* Myc-Mondo Complex (MML-1:MXL-2) Is Required for Resistance to Oxidative and Thermal Stress

Stress resistance is intimately connected to aging. For instance, reactive oxygen species cause the accumulation of oxidative damage to proteins and other cellular constituents as an organism ages [46]. Thermal stress also challenges the molecular chaperone network, leading to the collapse of proteostasis and a shortened lifespan [47]. Indeed, many of the genes known to influence longevity also function in stress response; the most studied example being ILS and DAF-16 (reviewed in [48]). DAF-16 functions in numerous forms of stress response including: heat shock, oxidative damage, exposure to heavy metals, radiation, anoxia, osmotic, and pathogen exposure [49]–[54]. Thus sensors of metabolic status or environmental damage may also regulate the expression of cytoprotective genes that influence aging. Based on the functional overlap between DAF-16 and Myc family members, we hypothesized that the Myc family influences longevity through altered stress response.

**Figure 6:**
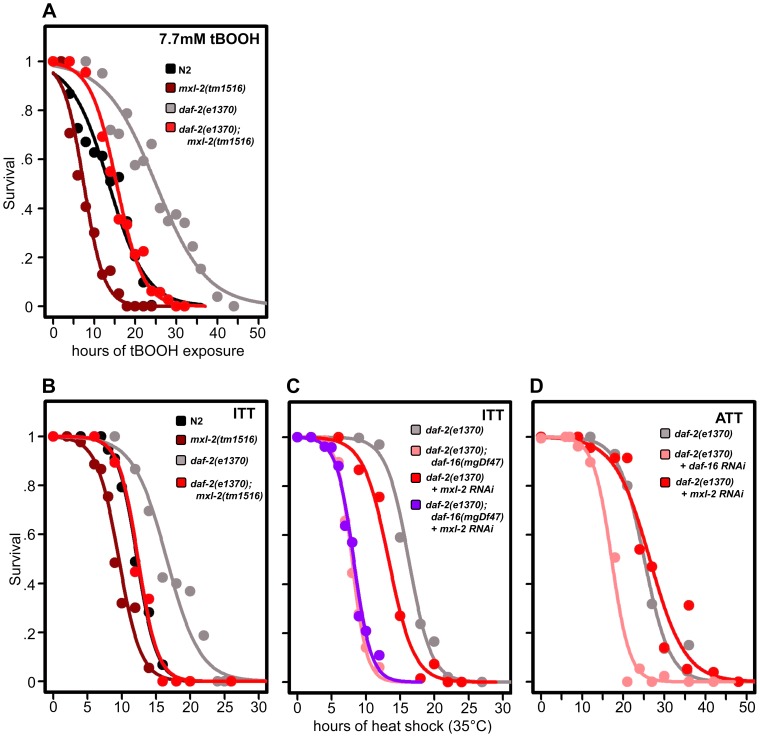
Loss of the Myc-Mondo complex impairs resistance to oxidative and thermal stress. (A) Loss of *mxl-2* impairs resistance to oxidative stress imposed by the exposure to *tert-*butylhydroperoxide (tBOOH) in wild-type and *daf-2(e1370)* mutant animals. Animals were exposed to 7.7mM tBOOH for the denoted time and survival was assessed. Loss of *mxl-2* in wild-type and *daf-2(e1370)* mutant animals impaired tBOOH survival to a similar extent. (B) Loss of *mxl-2* significantly impairs intrinsic thermotolerance (ITT) in wild-type and *daf-2(e1370)* mutant animals. (C) Loss of *mxl-2* does not further impair ITT in *daf-2(e1370);daf-16(mgDf47)* mutants. (D) *mxl-2* is dispensable for acquired thermotolerance in *daf-2(e1370)* mutants. Graphs were generated from the combined data from multiple experiments performed as described in Materials and Methods. Statistical analyses and experimental details can be found in Dataset S3.

To assess whether loss of the Myc-Mondo or Mad complex altered *C. elegans* oxidative stress resistance, N2, *mxl-2(tm1516)*, and *mxl-1(tm1530)* mutants were grown until day 2 of adulthood and survival was measured following oxidative stress imposed by exposure to tert-butylhydroperoxide (tBOOH; an organic peroxide). While loss of *mxl-1* failed to improve survival under any conditions (data not shown), loss of the Myc-Mondo complex via deletion of *mxl-2* impaired survival to oxidative stress (Figure 6A, Dataset S3). Specifically, following exposure to 7.7mM tBOOH, median survival was reduced from 13.6+/–0.8 hours in N2 animals, to 7.5+/–0.5 hours in *mxl-2(tm1516)* animals, a reduction of 45% (Figure 6A, Dataset S3). As expected, *daf-2(e1370)* mutant animals were much more resistant to tBOOH treatment; median survival was 25.0+/–1.0 hours. However, the median survival of *daf-2(e1370);mxl-2(tm1516)* double mutant animals was only 15.5+/–0.6 hours, a reduction of 38% (Figure 6A, Dataset S3). Thus loss of *mxl-2* does not reduce *daf-2(e1370)* tBOOH survival to a greater extent when compared to N2 (p = 0.986). To our surprise, *eat-2(ad465)* mutant animals were significantly more sensitive to tBOOH treatment than wild-type animals (data not shown), precluding a requirement to test the effects of *mxl-2* deletion in this background. Thus the Myc-Mondo complex is essential for resistance to oxidative stress and loss of the Mad complex is insufficient to increase stress resistance. Our bioinformatic analysis of oxidative stress response genes found that 12 out of 50 genes annotated as responding to oxidative stress are bound by DAF-16 and MDL-1 (i.e. no overrepresentation vs. the genome, p = 0.088). In contrast, 20 out of 50 oxidative stress response genes are bound by DAF-16, a significant enrichment (p = 0.049). Taken together, these data suggest that the *C. elegans* Myc-Mondo complex is generally required for resistance to oxidative stress, rather than specifically required for the enhanced resistance of the *daf-2(e1370)* mutant.

We next assessed whether loss of the Myc-Mondo or Mad complexes altered *C. elegans* thermotolerance (i.e. intrinsic thermal tolerance, ITT). N2, *mxl-2(tm1516)*, and *mxl-1(tm1530)* mutants were grown until day 2 of adulthood and survival at 35°C was measured. Loss of *mxl-2* significantly shortened the median survival time of wild-type animals by 22.0%, from 12.3+/–0.4 hours to 9.6+/–0.3 hours at 35°C (Figure 6B, Dataset S3, p<0.0001). Similar results were obtained in the *mml-1(ok849)* mutant (data not shown). Nearly identical results were obtained in the *eat-2(ad465)* and *eat-2(ad465);mxl-2(tm1516)* animals (Figure S5) confirming previous reports that *eat-2* mutations do not enhance thermal stress resistance [55]. Conversely, loss of *mxl-1* failed to alter survival to thermal stress (Dataset S3) in all three genetic backgrounds.

As previously reported, *daf-2(e1370)* mutant animals had increased thermal stress resistance compared to N2 animals (median survival of 16.6+/–0.8 hours versus 12.3+/–0.4 hours, Figure 6B, 6C, Dataset S3), and *daf-16* was essential for this increased resistance (Figure 6C, Dataset 3, and [56]). *daf-2(e1370);mxl-2(tm1516)* had thermal stress resistance comparable to wild-type control animals (median survival of 12.4+/–0.5 and 12.3+/–0.4 hours, respectively) (Figure 6B, 6C). Thus loss of Myc-Mondo function suppresses the enhanced survival of *daf-2(e1370)* mutant animals and wild-type survival to similar extents (25.3% and 22.2%, respectively).

We sought to better understand the relationship between ILS, DAF-16, the Myc family of transcription factors, and thermal stress resistance. To this end we first tested whether inactivating *mxl-2* in the absence of *daf-16* would further impair survival to thermal stress. Inactivating *mxl-2* in *daf-2(e1370);daf-16(mgDf47)* resulted in no significant further reduction in thermal stress survival when compared to control (median survival 8.0+/–0.11 and 7.6+/–0.10, Figure 6C, p = 0.313). We next asked whether *mxl-2* was required for the *acquired* thermotolerance (ATT) of *daf-2(e1370)* mutant animals. *daf-2(e1370)* mutant animals grown at 25°C become extremely tolerant to thermal stress. By day 3 of adulthood *daf-2(e1370)* animals subsequently exposed to 35°C have a median survival of 25.1+/–0.6 hours (Figure 6D), which is significantly greater than *daf-2(e1370)* intrinsic thermotolerance (ITT) (p<0.0001). Similar to ITT, the acquired thermotolerance of *daf-2(e1370)* is dependent upon *daf-16,* as median survival is reduced from 25.1+/–0.6 to 17.2+/–0.53 hours (Figure 6D). In contrast, *mxl-2* is dispensable for the acquired thermotolerance of *daf-2(e1370)* as median survival is unchanged (Figure 6D, p = 0.17). A comparison of genes that have promoter regions bound by DAF-16, PHA-4, and MDL-1 against all genes in the genome revealed a 2.17-fold enrichment (p = 0.006) for genes associated with a “response to heat”; which includes chaperones such *sti-1, hsp16.2, and hsp-16.41*. Collectively, we conclude that the Myc-Mondo complex: is required for intrinsic but not acquired thermotolerance; has overlapping genetic requirements with *daf-16;* and that MDL-1 and DAF-16 bind at the promoters of molecular chaperones implicated in the heat shock response.

### *C. elegans* Mad and Myc-Mondo Complexes Have Opposing Roles in Proteostasis

We determined whether loss of *C. elegans* Myc-Mondo or Mad complexes might alter the decline/progressive collapse of protein homeostasis (‘proteostasis’), a hallmark of normal aging [57]. One way to assess whether a genetic perturbation affects *C. elegans* proteostasis is by monitoring the solubility of a polyglutamine repeat fused to YFP (*Punc-54::Q35::YFP*). During normal aging transgenic animals expressing such poly Gln-YFP fusion proteins in body wall muscle cells accumulate protein aggregates that can be visualized as fluorescent foci [58]. Later in life, these toxic foci overwhelm the chaperone network that maintains proper protein folding, resulting in the collapse of proteostasis within the body wall muscle cells, thus causing paralysis in the animal [59].

**Figure 7:**
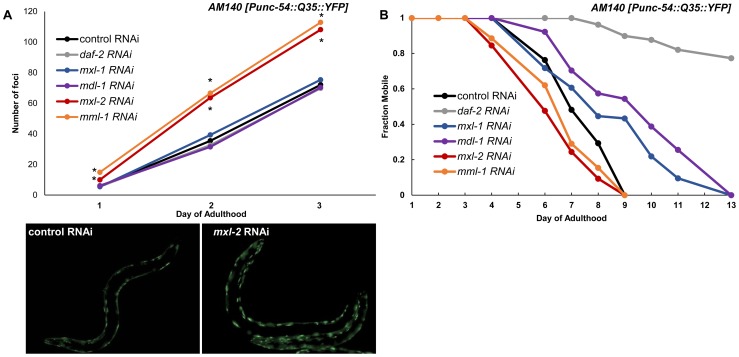
Myc-Mondo/Mad transcription factors influence proteostasis. (A) RNAi inactivation of *mxl-2* and *mml-1* leads to early accumulation of Q35::YFP foci (red and orange versus black). RNAi inactivation of *mxl-1, mdl-1,* and *daf-2* fail to delay accumulation of Q35::YFP foci (compare vector control RNAi treated animals (black) to blue, purple, and gray respectively. Traces are the combined data from three trials (*denotes p<0.01, standard deviations and statistical analysis can be found in Dataset S3). Images are from empty vector control and *mxl-2* RNAi treated animals of Day 2 of adulthood. (B) RNAi inactivation of *mxl-2* and *mml-1* leads to premature paralysis (compare red and orange to vector RNAi control (black)). RNAi inactivation of *mxl-1(blue), mdl-1(purple),* and *daf-2(gray)* delays paralysis induced by *Q35::YFP* expression in the body wall muscles in comparison to animals treated with empty vector control RNAi (black). Traces are representative of data from two trials performed in duplicate. Complete statistical analysis for data represented in this figure can be found in Dataset S3.

To test whether the Myc-Mondo and Mad complexes might function in proteostasis we inactivated *mml-1*, *mxl-2*, *mdl-1*, or *mxl-1* in *Pmyo-3::Q35::YFP* animals and quantified the accumulation of fluorescent foci (protein aggregates) over time. Loss of the Myc-Mondo complex (either *mml-1* or *mxl-2*) resulted in a significantly premature accumulation of protein aggregates (*mxl-2:* day 1 p = 7.6×10^−4^, day 2 p = 4.21×10^−6^, day 3 p = 9.66×10^−6^; *mml-1:* day 1 p = 5.68×10^−10^, day 2 p = 2.89×10^−9^, day 3 p = 5.86×10^−8^). For instance, by day 3 of adulthood *mml-1(RNAi)* or *mxl-2(RNAi)* treated *Q35::YFP* animals showed on average 113+/–16.7 and 108+/–22.1 foci protein aggregates, respectively. In contrast, control RNAi animals had an average of 72+/–13.6 foci per animal (Figure 7A, Dataset 3). This is consistent with the notion that loss of the Myc-Mondo complex compromises the protein chaperone network. In contrast, inactivation of the Mad complex (*mdl-1* or *mxl-1*) had no effect on polyglutamine foci formation (Figure 7A, Dataset 3). For example, at day 3 adulthood, *Q35::YFP* animals treated with *mdl-1* or *mxl-1* RNAi had on average 70+/–11.7 and 75+/–10.4 foci, which is not significantly different from animals fed control RNAi (*mdl-1* p = 0.711, *mxl-1* p = 0.458). Of note, inactivation of *daf-2* also failed to appreciably delay the accumulation of foci (Figure 5A): the average number of foci at day 3 adulthood is 70+/–18.0 (p = 0.791). Thus loss of the Mad complex or decreased ILS does not appreciably alter the acute accumulation of protein aggregates in *Q35::YFP* animals, but loss of the Myc-Mondo complex hastens the collapse of proteostasis as measured by the accumulation of fluorescent aggregates.

We assessed the long term consequences of loss of Myc-Mondo or Mad complex function in *Punc-54::Q35::YFP* animals as measured by the onset of paralysis. *daf-2(RNAi)* served as a positive control and completely negated the onset of paralysis over the course of our analysis, consistent with published results [60]. In contrast, the average age of paralysis of *Q35::YFP* animals treated with control RNAi was day 7 of adulthood, and all animals were paralyzed by day 9. Inactivation of *mml-1* or *mxl-2* resulted in a significant premature onset of paralysis *(mxl-2* p = 0.0000 and *mml-1* p = 4.93×10^−11^, Figure 7B). Conversely, inactivation of *mdl-1* or *mxl-1* resulted in a significant (*mxl-1* p = 0.0187; *mdl-1* p = 0.0384) delay in the onset of paralysis (Figure 7B). Collectively, our findings indicate that the *C. elegans* Myc-Mondo and Mad complexes have opposing effects on proteostasis, which parallel their effect on longevity and known roles in transcriptional control [11].

## Discussion

**Figure 8:**
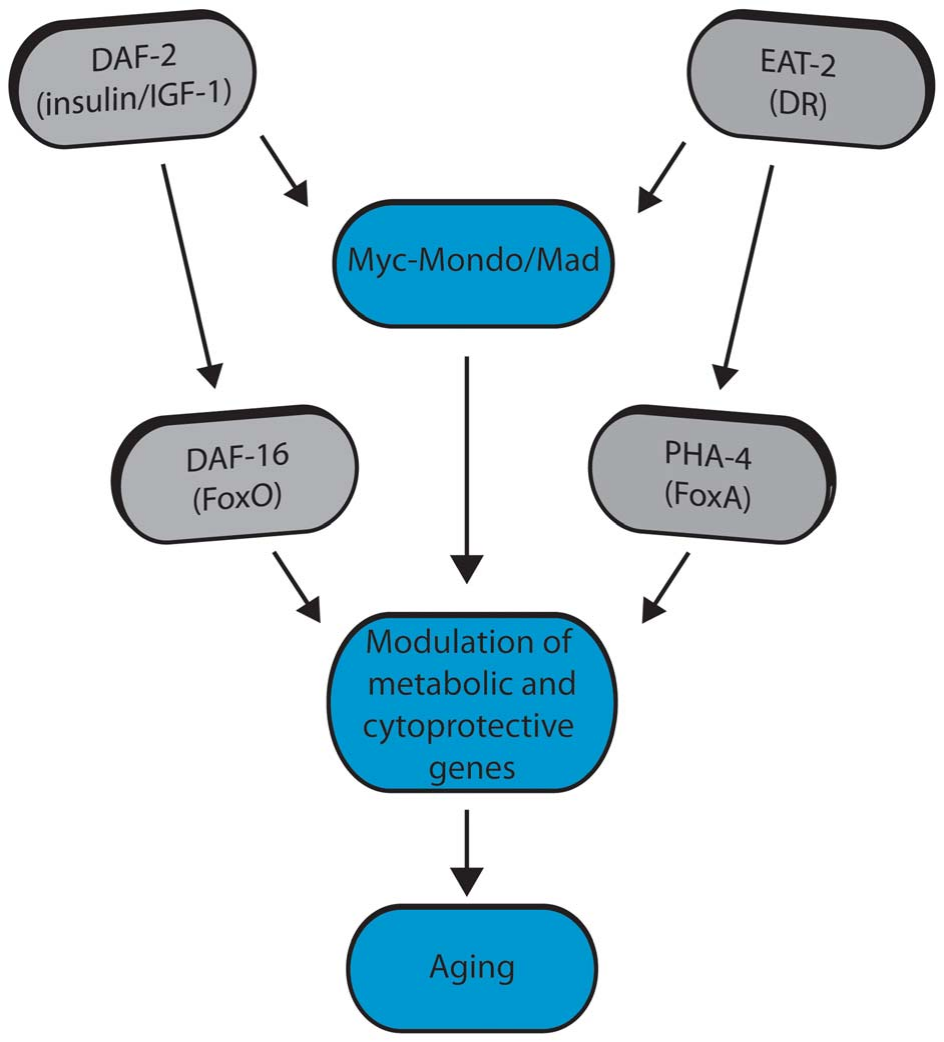
Model for Myc-Mondo/Mad transcription factors in longevity control under basal conditions the Myc-Mondo activation complex (MML-1:MXL-2) is largely inactive, and transcription of genes encoding functions related to aging is limited by the Mad transcriptional repression complex (MDL-1:MXL-1). Conditions of reduced ILS and DR promote Myc-Mondo complex activity, likely by regulating cellular localization and transcription of *mml-1.* Myc-Mondo/Mad transcription factors may cooperate with DAF-16 and PHA-4 to modulate the expression of key metabolic and cytoprotective genes to influence aging.

The Myc family of transcription factors have well established functions in growth control and metabolic regulation; processes that are known to influence lifespan. Yet, this is the first study to identify a role for the Myc interaction network in longevity control and proteostasis. We have identified a role for the Myc-family of bHLH transcription factors: *mml-1, mdl-1, mxl-1,* and *mxl-2* in the regulation of *C. elegans* lifespan. We show that the Myc-Mondo (MML-1:MXL-2) and Mad (MDL-1:MXL-1) transcriptional complexes have opposing roles in longevity control and proteostasis analogous to their known functions as transcriptional activators and repressors in *C. elegans* [11]. Specifically, loss of the Myc-Mondo complex leads to premature aging, while loss of the Mad complex delays aging. Lifespan extension by loss of the Mad complex is dependent upon the Myc-Mondo complex, *daf-16*, and *pha-4,* suggesting a common mechanism in longevity control. Conversely, Myc-Mondo is required for lifespan extension by decreased ILS or conditions of DR. Both decreased ILS and conditions of DR promote nuclear accumulation of MML-1, but do so through distinct mechanisms; altered DAF-16 activity regulates expression of Myc family members, while *pha-4* is essential for the increased nuclear accumulation of MML-1. In contrast, the Myc-Mondo complex is dispensable for the nuclear accumulation of DAF-16 by decreased ILS, and overexpression of DAF-16::GFP rescues the shortened lifespan of *mxl-*2 null mutant animals, which suggests that DAF-16 and the Myc-Mondo complex may co-regulate transcription at overlapping target genes. From a candidate approach, we find that Myc-Mondo is required for the induction of longevity genes by decreased ILS. DAF-16, PHA-4, and MDL-1 bind within the genome at many overlapping target genes involved in unfolded protein binding, carbohydrate metabolism, autophagy, and stress response. Finally, loss of the Myc-Mondo complex impairs oxidative and thermal stress survival. Collectively, our results suggest that Myc family members are regulated by diverse signals of metabolic status and converge with DAF-16 and PHA-4 at metabolic and cytoprotective transcriptional programs that influence aging (Figure 8).

The Myc interaction network in mammals has been defined based on the combinations of known protein-protein interactions between Myc/Mnt/Mad/Mondo and either Max or Mlx, which distinguishes them from the larger super-family of bHLH transcription factors [61]–[64]. The discovery that the Myc-Mondo and Mad complexes have antagonistic functions in both transcription and longevity control sets them apart from other transcription factors that are relevant to aging, such as DAF-16, PHA-4, or SKN-1. The related bHLH protein, MXL-3, has recently been implicated in lifespan [23], [65]. *mxl-3* encodes a paralog of *mxl-1* (Max), which is unique to nematodes [22]. Furthermore, MXL-3 homodimerizes and does not interact with other *C. elegans* Myc family members [10]. This is in contrast to mammalian Max and *C. elegans* MXL-1 (Max), which function as heterodimers. Additionally, there is little overlap between predicted MXL-3 and MDL-1::MXL-1 target genes [10]. In *C. elegans*, MXL-3 functions in conjunction with HLH-30 to regulate the expression of a family of lipases, which mobilize lysosomal fat stores, and function independent of ILS and DR to influence longevity [23], [65]. We find no genetic interaction between the *C. elegans* Myc-Mondo or Mad complexes and *mxl-3,* implying that MXL-3 functions are distinct from the function of the Myc-Mondo/Mad interaction network.

MML-1, (for ““Myc and Mondo-like”), has a high degree of sequence identity to c-Myc, MondoA, and MondoB/ChREBP. The *C. elegans* genome does not encode a clear Myc ortholog [22]. Therefore, it is likely that *C. elegans* MML-1 incorporates both mammalian c-Myc and Mondo functions. Myc proteins have been implicated in ribosomal and mitochondrial biogenesis, energy metabolism, biosynthesis, cell growth, and cell cycle regulation. For example, many glucose metabolism genes are directly regulated by Myc [66]–[71]. In this way, Myc promotes glucose transport and catabolism. *C. elegans* MML-1 has an overall domain structure and size similar to mammalian Mondo/ChREBP. Work in mammals has identified MondoA:MLX and ChREBP:MLX complexes as non-hormonal sensors of glucose and key regulators of glycolytic metabolism, fat storage, and energy sensing [20], [72]. Recently, ChREBP has been shown in adipose tissue to regulate systemic glucose metabolism, fatty acid synthesis, and insulin sensitivity [73]. While the genomic binding patterns of MML-1 remain to be determined, we find an overrepresentation of genes involved in carbohydrate metabolism bound by MDL-1, DAF-16, and PHA-4. Thus the Myc-Mondo/Mad complex functions relevant to longevity may be at least partially linked to evolutionarily conserved alterations in carbohydrate metabolism.

Surprisingly, there have been few large-scale efforts to identify Mad targets in mammalian systems. Individual Mad target genes have largely been identified by the examination of genes previously identified as Myc targets [40]. However, one study overexpressed Mad1 in T-lymphocytes and identified reduced expression in 57 genes. [39]. Collectively, of 64 putative Mad1 target genes we found 53 clear *C. elegans* homologues, of which 41 are MDL-1 targets (Figure 5, Dataset S2). Many of these homologues are components of important cellular processes such as protein translation/ribosome function (23), metabolism (7), DNA transcription (2), and cell cycle control (2). Moreover, 11 have been implicated in *C. elegans* longevity: the eukaryotic initiation factors *eif-1/*eIF1, *ifg-1/*eIFGI, *ife-2/*eIF4E, and *inf-1/*eIF4AI*;* elongation factors *eef-1a.2/*EF1 and *eef-2/*EF2; the ribosomal protein *rpl-18/*Rpl18; the PTEN homologue *daf-18*; the glucose-6-phosphate isomerase *gpi-1*; the mitochondrial stress protein *hsp-6*; and the cytochrome b-c1 complex subunit *ucr-1*. Thus while the full extent of *bona fide* Mad target genes in mammals is relatively unknown, we find good overlap at conserved putative target genes, suggesting that the expression of Mad target genes relevant to longevity may be conserved.

We have found that decreased ILS and conditions of DR regulate *C. elegans* Myc family activity. Both decreased ILS and conditions of DR promote nuclear accumulation of MML-1, presumably to alter gene expression. In this respect MML-1 resembles its mammalian homologues, MondoA and ChREBP, which are also regulated by cytoplasmic-to-nuclear shuttling under changing conditions of nutrient availability. In contrast to mammalian Mondo complexes, Mad complexes are reported to be constitutively nuclear [74], [75]. Furthermore, we find that DAF-16 activity regulates Myc family expression. This is reminiscent of the reported function of mammalian *MAD1,* which is upregulated at the transcriptional level by several different signaling pathways, including activated PI3K/Akt downstream of the granulocyte-colony stimulating factor receptor (G-CSFR*)* [76]. This contrasts with findings that identified *mdl-1* as an upregulated DAF-16 target gene by microarray analysis [13], [77]. Furthermore, a previous study found that *mdl-1* inactivation slightly shortened *daf-2* mutant longevity [13]. We find no effect of loss of *mdl-1* on *daf-2(e1370)* longevity (data not shown). One possible explanation is that the published lifespan analysis was conducted at 25°C, which we find is a slightly stressed state that alters the genetic requirements for lifespan extension by decreased ILS (A.V.S. unpublished observations).

We found that the Myc-Mondo and Mad complexes function in both DR and ILS signaling, implying that the Myc-Mondo/Mad complexes may act as a molecular convergence point to integrate cues of metabolic status, and coordinate the transcriptional response to these diverse signals. A number of metabolic signals are widely accepted as being intimately linked to aging, yet how these signals are integrated to “fine-tune” the appropriate transcriptional response to maximize longevity under adverse environmental conditions is unknown. Interestingly, Myc has recently been shown to function as a “universal amplifier” of transcription; instead of having distinct target genes, Myc amplifies the output of the existing gene expression [78], . Thus the Myc-Mondo and Mad complexes’ antagonistic effect on transcription may function as a rheostat to modulate gene expression in response to multiple inputs of metabolic status, which is a possibility mutually exclusive from regulating transcription of distinct target genes.

Our results raise a number of questions of how Myc family members integrate diverse signals of metabolic status. The roles of ILS and DR in longevity control are genetically separable, yet there is evidence of overlap between the transcriptional programs that are activated by decreased ILS and conditions of DR (Reviewed in [1]). For instance, overexpression of *pha-4* has the greater influence on lifespan in the absence of *daf-16* [8], which suggests either an inherent competition between *daf-16* and *pha-4* in wild-type animals, or that the role of *daf-16* and *pha-4* may be partially redundant. However, *pha-4* is dispensable for the increased lifespan of *daf-2* mutants [8], yet wefind that *pha-4* is necessary for the increased nuclear accumulation of MML-1::GFP by decreased ILS. One relevant finding is that loss of *pha-4* has no detrimental effect on the distribution of MML-1::GFP localization in wild-type animals, which still have substantial amounts of nuclear MML-1. Thus the basal distribution of MML-1 may be sufficient to potentiate insulin/IGF1 signals either through altered patterns of DNA binding, recruitment of co-factors, or changes of stability at DNA. However, our results predict that there would be no additivity of lifespan extension by the combination of DR and decreased ILS in the absence of *pha-4.* The mechanism through which PHA-4 potentiates DR signals through increased nuclear accumulation of MML-1 remains to be determined. Nuclear localization of ChREBP, the mammalian homologue of MML-1, is regulated by changes in carbohydrate availability through post-translational modifications [80]. Thus identifying whether MML-1 is similarly regulated will be an important step to further understand how DR signaling alters Myc family function.

We find that overexpression of DAF-16 can rescue the shortened lifespan caused by the loss of Myc-Mondo, yet Myc-Mondo is required for the increased expression of DAF-16 target genes under conditions of decreased ILS. How might these two paradoxical findings be resolved? One possibility is that DAF-16 activity is not maximally induced by *daf-2(e1370).* Our discovery that the *daf-16* dependent acquired thermotolerance of *daf-2(e1370)* is independent of *mxl-2* is consistent with the possibility that mild heat stress in *daf-2(e1370)* maximally potentiates DAF-16 activity, thereby removing the need for the Myc-Mondo transcriptional activation complex. A second possibility is that DAF-16::GFP overexpression is not equivalent to the activation of DAF-16 in *daf-2(e1370)* mutant animals.

We sought to identify putative common target genes between DAF-16, PHA-4, and MDL-1 to begin to elucidate the molecular mechanisms through which the Myc family may influence longevity. In contrast to DAF-16/PHA-4, MDL-1 and the Mad mammalian homologues are only known to function as transcriptional repressors [11], [40]. Importantly, MDL-1 genomic binding patterns were identified in wild-type animals; thus under normal conditions the MDL-1 complex limits the expression of DAF-16/PHA-4 target genes relevant to aging. We favor a model where under conditions of reduced ILS or DR, increased nuclear accumulation of MML-1 results in the replacement of MDL-1 binding at target genes. This idea is consistent with the model of complex switching that has been proposed to describe the antagonistic relationship between Myc and Mad complexes in mammals [40]. We found significant overlap of genomic binding between these transcription factors at many target genes, consistent with the possibility of shared transcriptional programs. Assessing whether these putative common target genes were enriched over the genome for any specific biological function by gene ontology revealed a significant enrichment for genes that function in lifespan (299), stress response (117), carbohydrate metabolism (93), unfolded protein binding (27), and autophagy (9). Specific forms of stress response that were over-represented included response to heat (12), unfolded protein response (20), immunity/pathogen resistance (15), and DNA damage response (49). Surprisingly, response to oxidative stress was not over-represented. However, we found no evidence that heat or oxidative stress altered the expression of Myc family members, nor the nuclear accumulation of MML-1 (D.W.J., unpublished observations). Thus despite their requirement for stress response survival, and the finding that MDL-1 binds at many genes involved in survival to stress, Myc family members do not seem to be stress response genes, which is in contrast to their regulation by altered signals of metabolic status. Rather, these data support a model where the Myc family converges with DAF-16 and PHA-4 at overlapping metabolic and cytoprotective transcriptional programs that set the progression of aging.

Given their close ties to metabolism it is not surprising that DAF-16 and PHA-4 bind extensively in the promoter regions of key metabolic genes. Our analysis shows that MDL-1, DAF-16, and PHA-4 binding is significantly enriched at genes involved in carbohydrate metabolism (Dataset S2), including nine glycolytic genes (Y77E11A.1, H25P06.1, *enol-1*, *pyk-2*, ZK632.4, *gpi-1, aldo-2,* F57B10.3, *pgk-1*), and five gluconeogenesis genes (*gpi-1, aldo-2,* F57B10.3, *pgk-1, pyc-1*), which is strongly reminiscent of mammalian Mondo-ChREBP/Mlx complexes [18]-[20], [42]. Interestingly, it has been found that gluconeogenesis is upregulated in conditions of low ILS and DR [44], [81]; therefore, it is possible that one role for Myc-Mondo complexes under these conditions is to promote the expression of gluconeogenic genes, which results in the increase of intracellular glucose. Other metabolic genes with known roles in longevity included *icl-1* and *stdh-1*. Both are metabolic enzymes that are upregulated by decreased ILS, in a *daf-16* dependent manner [37]. We discovered their upregulation by decreased ILS is also dependent on the Myc-Mondo complex (Figure 5F). *stdh-1* encodes a steroid dehydrogenase, which is involved in hormone biosynthesis and suggests that the Myc family may contribute to cell non-autonomous signaling. *icl-1* encodes a dual enzyme isocitrate lyase/malate synthase essential for the glyoxylate cycle, a variation of the citric acid cycle that allows the production of sugar from fat. Like gluconeogenesis, the expression of *icl-1* is also upregulated in conditions of low ILS and DR [13], [44], and increased glyoxylate cycle activity is required for the increased longevity of *C. elegans* insulin signaling mutants [13].

Proteostasis is the ability of the cell’s proteome to maintain a proper folding environment. The predominant theory is that during aging, post-mitotic cells accumulate protein damage (e.g. through oxidative damage and DNA mutations), which in turn produces misfolded proteins that rapidly form highly stable protein aggregates that ultimately overwhelm the ability of the chaperone network, protein degradation machinery, and sequestration strategies to maintain a proper folding environment. This collapse may be the underlying basis for age-associated proteotoxic diseases [82]. We find that the Myc-Mondo and Mad complexes have opposite effects on proteostasis, analogous to their effect on transcription and longevity. Since many putative target genes of MDL-1 are involved in either protein folding, unfolded protein response, or heat-shock response, we favor a model where changes in Myc family activity alter the expression of genes involved in protein homeostasis, which in turn influences the progression of aging.

Putative target genes of MDL-1 and DAF-16 were enriched over genes bound only by DAF-16 in two major gene ontology terms that are highly relevant for the maintenance of proteostasis: unfolded protein binding and the unfolded protein response. These somewhat mutually exclusive categories cover *de novo* protein folding and the response to proteotoxic stress, respectively. Twenty-eight of 52 genes annotated to function in unfolded protein binding were discovered and include the gene classes: *cct* (Chaperonin Containing TCP-1; 6 of 8), *pfd* (PreFolDin; 4 of 6), and *dnj* (DNaJ domain; 11 of 29) (Dataset 3). The *cct* genes encode the components of the TCP-1 ring complex (TRiC), which complete *de novo* protein folding and interacts with prefoldin co-chaperones. The TRiC complex has been implicated in longevity control and an ILS-mediated soma to germline transformation gene expression program [83], [84]. In addition, 20 of 40 genes annotated to function in the unfolded protein response are bound by DAF-16, PHA-4, and MDL-1. Genes involved in the mitochondrial and ER unfolded protein responses were represented (3 and 9 genes respectively, Dataset S2). Lifespan extension in *daf-2* mutants is dependent on both *ire-1* and *xbp-1*, suggesting that genes involved in ER proteostasis may contribute to *daf-2* mutant lifespan [85]. We found several small heat shock proteins (*hsp-16.2, hsp-16.41,* and *hsp-12.6)* to be bound by DAF-16, PHA-4, and MDL-1. The latter is not stress responsive, but is upregulated in *daf-2* mutant animals dependent on *daf-16* [13], [86], [87], and we find this upregulation is also *mxl-2* dependent (Figure 5D). Interestingly, *hsp-12.6* is required for the increased lifespan of ILS mutants, and *hsp-12.6(RNAi)* results in the premature collapse of proteostasis [88].

This is the first study to identify a role of the Myc-Mondo/Mad interaction network in longevity. Significantly, a homologue of one of these transcription factors may also influence aging in mammals. The *mml-1* mammalian homologue ChREBP is also known as William-Beuren Syndrome Chromosomal Region 14 (WBSCR14). Williams-Beuren Syndrome (WBS) is a multisystem disorder resulting from the deletion of 26 to 28 genes, including ChREBP, on human chromosome 7. WBS has been characterized as a disorder of mild accelerated aging. Patients display premature gray hair, diverticulosis, diabetes, and hearing loss that develops during adolescence or young adulthood, in combination with instances of declining memory skills or dementia [21]. Thus our findings collectively imply that the Myc-Mondo/Mad interaction network of basic-helix transcription factors function as a molecular convergence point: integrating diverse signals of metabolic status that converge at evolutionarily conserved metabolic and cytoprotective transcriptional programs to influence the progression of aging.

## Materials and Methods

### Strains, Plasmid Construction, RNAi, and Nematode Culture

Standard culture techniques were used to maintain nematodes [89]. The wild-type strain is Bristol N2 and all other strains used in this work were backcrossed into Bristol N2 a minimum of six times before use. The *mxl-2(tm1516)*, *mxl-1(tm1530)*, and *mdl-1(tm311)* strains were created by and obtained from Dr. Shohei Mitani with the Japanese Bioresource Project; the *mml-1(ok849)* and *mxl-3(ok1947)* were generated by Dr. Robert Barstead with the Oklahoma Medical Research Foundation Knockout Group, and were generously provided by the *C. elegans* Genetics Center (University of Minnesota).

The *Pmml-1::mml-1::gfp* construct was generated through PCR sewing [90]. The *mml-1* genomic fragment was PCR amplified from the WRM0615CE07 fosmid using the following primers: 5′-AGTCGACCTGCAGGCATGCAAGCTaaatggatttttgagttgttgcat-3′ and 5′-TAGAGGGTCGCGTTAGAGGT-3′. The GFP open-reading frame was PCR amplified from pPD95.75 (Fire Lab vector kit) using the following primers: 5′-AGCTTGCATGCCTGCAGGTCG-3′ and 5′-AAGGGCCCGTACGGCCGACTA-3′. The two fragments were then PCR sewn together using the following primers: 5′-TTCCACTCGTTTCTCCGTCC-3′ and 5′-GGAAACAGTTATGTTTGGTATA-3′. Eight sewing reactions were simultaneously performed. Each reaction generated a single PCR product, which were combined to form a single pool of DNA which was injected into *mml-1(ok849)* young adult hermaphrodites at a concentration of 5 ng/μL along with 2.5 ng/μL pCFJ90 as a co-injection marker, and 142.5 ng/μL 2-log DNA ladder (New England Biolabs). The transgene was maintained as a high-transmission rate extrachromosomal array. Three independent *mml-1(ok849)* lines expressing this array were subsequently mated into *daf-2(e1370)* and *eat-2(ad465)* mutant to generate independent lines for examination. Three independently generated lines were examined per background.

The strains used in this work are: N2 Bristol, eat-2(ad465), daf-2(e1370), mxl-2(tm1516), mxl-1(tm1530), mdl-1(tm311), mml-1(ok849), mxl-3(tm1947), mxl-2(tm1516);daf-2(e1370), mxl-1(tm1530); daf-2(e1370), mxl-2(tm1516);eat-2(ad465), mxl-1(tm1530);eat-2(ad465), AM140 rmIs132 [Punc-54::Q35::YFP] [58]; AVS317-319 mml-1(ok849);artEx6-8 [Pmml-1::mml-1::gfp; pCFJ90] (three independent lines), AVS329-331 daf-2(e1370);artEx6-8 [Pmml-1::mml-1::gfp; pCFJ90] (three independent lines), AVS332-334 eat-2(ad465);artEx6-8artEx3 [Pmml-1::mml-1::gfp; pCFJ90] (three independent lines). RNAi clones were grown overnight in Luria broth and seeded onto plates containing 5 mM isopropylthiogalatoside, to induce dsRNA expression overnight at room temperature.

### Lifespan Assays

Lifespan and survival assays were performed using either a replica set protocol, as previously described [12], or through traditional methods. Briefly, for replica set protocol: rather than following a single population of animals over time, animals were cultured in 24-well format on HT115 bacteria expressing either empty vector or the indicated RNAi. A sufficient number of plates were created for each condition (combination of strain and RNAi) so that each could be counted at least every other day and then disposed. On average, 25 animals were counted per well. In all cases animals were cultured from L1 to L4 stage of development at 15°C, at which point FuDR was added to a final concentration of 400 μM and the animals were transferred to 20°C for the remainder of their lifespan. For traditional survival assays, proportional survival was charted over time using the Kaplan-Meier estimator. Replicate survival assays were fit via generalized linear modeling to a logit curve, according to the equation y = 1/(1+e∧(a*x-b)). The LD50 for a fit survival curve was then a function of the coefficients: LD50 = b/a. For two sample comparison, p-values were calculated through resampling and refitting, where the difference in resampled LD50s was compared to the difference in the observed LD50s, see [12]. Similar analyses were used on stress survival assays. For each comparison of either a mutant or RNAi treatment compared to control, between two and four independent trials were conducted to measure alterations in lifespan or survival (see figure legends and Dataset S1). Raw mortality data from replicate set lifespan has been deposited into the Dryad repository (doi:10.5061/dryad.pj0p3 Data files: Raw Data of Mortality Observations of Replica Set Experiments).

### MML-1::GFP Localization

Synchronized L1 transgenic animals were grown to the L4 stage of development on HT115 bacteria, expressing: empty vector control, *daf-16,* or *pha-4* RNAi at 15°C on 6 cm plates. FuDR was added to a final concentration of 400 μM and the animals were transferred to 20°C. GFP localization was examined on day 2 of adulthood. Worms were mounted on 2% agarose pads and anaesthetized with a mixture of 15 mM tricaine mesylate and 15 mM tetramisole hydrochloride prior to imaging. Microscopy was performed on a Zeiss AxioImager.M2m equipped with an AxioCam MRm camera using high NA Zeiss EC-Plan-NEOFLUAR 20X and 40X objectives, and a Semrock Brightline GFP filter (488 nm/535 nm Ex/Em). Scoring was performed in three separate trials examined under blinded conditions, and each animal was scored according to the rubric shown in (Figure 4).

### qRT-PCR

Synchronized populations were grown to young adulthood at 15°C on 10 cm RNA plates containing HT115 bacteria expressing vector control RNAi. Animals were then rinsed from the plates using M9 and washed three times to remove bacteria. RNA was isolated using Trizol reagent (Life Sciences) following the manufacturer’s protocol. Isolated RNA was converted to cDNA using the Bio-Rad iScript reverse transcriptase kit with oligo dT primers. Negative samples were prepared by omitting the reverse transcriptase enzyme from the reaction.

Analysis of transcript levels via qRT-PCR was performed on three distinct biological replicates per condition, and performed in triplicate in each instance. The total cDNA concentration for each sample was normalized to the levels of *act-1* and *rpl-32* transcript. *mml-1, mxl-2, mxl-1, and mdl-1* transcript levels were normalized to N2 control samples. For analysis of Class I genes: *act-1, rpl-32,* and *pat-10* were used to normalize total cDNA concentration. *dod-3, hsp-12.6, stdh-1,* and *icl-1* transcript levels were normalized to N2 control samples. Statistical analyses of fold-changes from three independent trials were accomplished using Student’s paired t-test.

The primers used are as follows:

*act-1*:forward 5′-CCA TCA TGA AGT GCG ACA TTG-3′

reverse 5′-CAT GGT TGA TGG GGC AAG AG-3′

*rpl-32:*forward 5′-AGG GAA TTG ATA ACC GTG TCC GCA-3′

reverse 5′-TGT AGG ACT GCA TGA GGA GCA TGT-3′

*mml-1:*forward 5′-GGA GAA ATC CGG AAG GCA TTA CTT AC-3′

reverse 5′-CGC CAG AAT ACA AAT CGG GTA TAA TAT C-3′

*mxl-2:*forward 5′-TCA GAG CCT GCG ACT TCA TG-3′

reverse 5′-GTC GAG GAG AAG TTG GAG CAT-3′

*mxl-1:*forward 5′-GAC ATG AGT GAC CTC GAA GAT GAC-3′

reverse 5′-CAG GCG AGC TAT CTC TTC TCT G-3′

*mdl-1:*forward 5′-CGA TCT TTC AAA TGA GTC CGA ACT CC-3′

reverse 5′-GTC TTG CAA TTC AAT GAT GTG ATC TCG-3′

*pat-10:* forward 5′-GACGGAAAGCTTCACGAAGTTC-3′

reverse 5′-CCTTCGTAAACTGATCCGCAAG-3′

*dod-3:* forward 5′-AAAAAGCCATGTTCCCGAAT-3′

reverse 5′-GCTGCGAAAAGCAAGAAAAT-3′

*hsp-12.6:* forward 5′-GGAGTTGTCAATGTCCTCGACG-3′

reverse 5′-GAAGTTCTCCAATGTTCTTGAC-3′

*stdh-1:* forward 5′-ACAGGATGTCTTCAAAAGGAATATGG-3′

reverse 5′-TTGCTGGGGTGATAGCTTGG-3′

*icl-1:* forward 5′-GACTACGAGGCTGGAAGAACGATTG-3′

reverse 5′- GTAGGCGAACATCTTGTCTGGGTAC-3′

### Statistical and Informatic Analysis

Statistical analysis of traditional lifespan analysis was performed by two sample tests, which used the Mantel-Cox log-rank statistic. For replica set lifespan and stress survival experiments, data were fit to a two parameter logit survival curve, a generalized linear model with a binomial link function. This curve models the probability of a worm's survival at a given time point t, according to the equation p = 1/(1+e∧(+ax-b)). The parameters a and b, which represent logit slope and intercept, are optimized by iteratively reweighted least squares. For a fit logit curve, the LD50 is b/a, or intercept divided by slope. For maximum life, we took the value of the logit curve where it dropped below 5% survival. We estimate 95% confidence intervals on the LD50 non-parametrically, through resampling of observations (K = 10,000), with replacement. For two sample tests comparing condition LD50s to control, we again used a resampling approach, where labels were swapped between conditions compared, then curves refit, and the resampled difference in LD50 was compared to the observed difference, (K = 10,000).

To compare the effect loss that *mxl-2* had in different backgrounds, we compared the ratio of *mxl-2* loss of function LD50 over control LD50 between backgrounds. To put confidence intervals on this ratio, and make two sample comparisons, we again used resampling to estimate the distribution (K = 10,000 for each of the four conditions).

The binding site locations for PHA-4, MDL-1, or DAF-16 were identified based on ChIP-seq data generated by the modENCODE project (www.modencode.org). ChIP-seq binding peaks for DAF-16, PHA-4, and MDL-1 were validated in [38]. Binding sites were assigned to specific genes based on location data from WormBase (www.wormbase.org) and then PHA-4 and MDL-1 binding sites were mapped relative to the transcriptional start site (TSS) of nearby open reading frames. Examination of genome-wide binding patterns revealed that MDL-1 and PHA-4 binding was most highly enriched between –700bp and +100bp relative to the TSS. Based on this we assessed the binding of PHA-4 and MDL-1 within this window relative to each gene in the genome. Genes bound by DAF-16 were identified in [37].

For characterizing ontologies among genes bound by MDL-1, we used a list of GO terms relevant to aging mined from WormBase version 210. To evaluate GO term enrichment between a set of bound genes and the genome we used the chi-squared test.

### Thermotolerance, Oxidative Stress, and Proteostasis Assays

For stress response assays, synchronized L1 animals were cultured on HT115 bacteria expressing the identified RNAi vector at 15°C until the L4 stage of development. FuDR was then added to a final concentration of 400 μM, and the animals were then cultured at 20°C unless otherwise stated. For thermotolerance assays, two day old adult animals were exposed to 35°C for the indicated time period, then removed and allowed to recover for 1–2 hours at room temperature before survival was then scored. For oxidative stress survival assays, two day old adult animals were treated with 7.7 mM *tert-*butylhydroperoxide (Alfa Aesar) for the indicated time period, then survival was scored. For proteostasis assays, foci formation in AM140 animals was scored on days one, two, and three of adulthood. Scoring was performed on a Zeiss M2BIO upright fluorescence microscope using a 10X objective and an EGFP filter set. Worms were mounted on 2% agarose pads and anaesthetized with a mixture of 15 mM tricaine mesylate and 15 mM tetramisole hydrochloride prior to imaging. For paralysis assays animals were scored in their ability to retreat from gentle nose touch. Paralyzed animals were removed, and scoring continued until the entire population had become paralyzed. Foci accumulation and paralysis were measured in three separate trials each. For stress response assays two separate trials were performed in duplicate.

## Supporting Information

Figure S1 Myc-Mondo/Mad complexes likely function as heterodimers to influence longevity. (A) RNAi inactivation of *mxl-1* fails to further extend the lifespan of *mdl-1(tm311)* mutants. (B) RNAi inactivation of *mdl-1* fails to further extend the lifespan of *mxl-1(tm1530)* mutants. (C) RNAi inactivation of *mml-1* fails to further shorten the lifespan of *mxl-2(tm1516)* mutants. (D) RNAi inactivation of *mxl-2* fails to further shorten the lifespan of *mml-1(ok849)* mutants. The lack of an RNAi effect in these mutant backgrounds is likely not due to inefficacy on the part of the RNAi as the same RNAis (same bacterial culture) significantly influenced the lifespan of N2 animals within the same experiment. Furthermore, all four mutant strains responded to other RNAi inactivations within the same experiment. For details please refer to Dataset S1. (E and F) RNAi inactivation of *mxl-2* fails to suppress the longevity of long-lived *mxl-3(ok1947)* mutants (pink versus green traces). Similar results were obtained for *mml-1* RNAi (Dataset S1). The experiments in E and F were conducted by the replica set and traditional method, respectively.
(TIF)

Figure S2 RNAi inactivation of *mxl-2* and *mml-1* shorten, and *daf-2 RNAi* extends, *daf-2(e1370)* mutant lifespan. (A) RNAi inactivation of *mxl-2* and *mml-1* significantly shorten lifespan to an extent that is similar to what is observed in *daf-2(e1370);mxl-2(tm1516)* mutant animals. This result corroborates the results in Figure 2B. (B) RNAi inactivation of *daf-2* in *daf-2(e1370)* mutant significantly extends longevity.
(TIF)

Figure S3 Loss of *mxl-1* fails to extend longevity in the absence of *daf-3* RNAi inactivation of *mxl-1* robustly extends the lifespan of N2 animals but has no effect on the *daf-3(tm4940)* mutant.
(TIF)

Figure S4 Loss of *mxl-2* fails to shorten longevity in a strain that over-expresses *daf-16* Lifespan was analyzed in animals expressing DAF-16::GFP translational fusion protein in the presence or absence of *mxl-2* or *mxl-1.* Loss of *mxl-2* significantly shortens the lifespan of wild-type animals (compare black and red lines); however, the lifespan of DAF-16::GFP expressing animals was identical in wild-type, *mxl-2(tm1516),* and *mxl-1(tm1530)* mutant backgrounds (compare grey, pink, and blue lines). This suggests that increased *daf-16* expression can compensate for the loss of the MXL-2:MML-1 complex and that loss of the MXL-1:MDL-1 complex is not required for increased longevity when high levels of DAF-16::GFP are present.
(TIF)

Figure S5 Loss of *mxl-2* suppresses thermotolerance in *eat-2(ad465)* mutants similar to what was observed in wild-type and *daf-2(e1370)* mutant backgrounds loss of *mxl-2* significantly weakens *eat-2* mutant animals’ ability to survive thermal stress.
(TIF)

Figure S6 Replica set and traditional methods for scoring lifespan produce similar results. (A) Traditional lifespan analysis of *mxl-2(tm1516)*, *mml-1(ok849)*, and *mxl-1(tm1530)* mutants (compare to Figures 1B and 1C). (B and C) Replica set and traditional lifespan analysis using RNAi against all four Myc-Mondo/Mad transcription factors confirm the efficacy of RNAi clones and both methods produce comparable results. The replicate set method surveys many independent observations of whether a worm is alive or dead, then derives the median longevity (i.e. each worm is assessed whether it is alive or dead once). In contrast, the traditional lifespan method directly measures the mean lifespan of a single population tracked longitudinally in time. Both methods give a similar read for median lifespan.
(TIF)

Figure S7 Pharyngeal pumping rates in *eat-2(ad465)* single and double mutants pharyngeal pumping rates in *eat-2(ad465)* mutants are significantly lower compared to N2 animals as previously described [26], [27]. Subsequent mutations in *mxl-2* and *mxl-1* do not alter pumping rates of *eat-2(ad465)* mutants.
(TIF)

Dataset S1 Complete lifespan data and select statistical analyses. This dataset contains all lifespan data discussed within this manuscript (replica set and traditional), as well as data from other experiments that support lifespan data represented in in-text and supplemental figures. This dataset also contains select statistical analyses to support the significance of the lifespan differences described in the text from Figures 1-3.
(XLS)

Dataset S2 Bioinformatics analyses of ChIP-seq data analyses of available ChIP-seq data from the modENCODE project. This file includes statistical GO term analyses, as described in Materials and Methods, of MDL-1, DAF-16, and PHA-4 binding, and the assignment of bound genes to metabolic and stress response pathways (Tabs 1-3). Complete information of transcription factor binding to enriched GO terms (Tabs 4-9). Comparison of known Mad1 target genes and MDL-1 binding to *C. elegans* homologues (Tab 10). Within the spreadsheets an ‘X’ denotes inclusion in a category and ‘-‘ denotes exclusion.
(XLS)

Dataset S3 Complete oxidative stress response, thermotolerance, and proteostasis data. This dataset contains all data pertaining to Figures 6 and 7: including LD50s, p-values, and total number of animals examined for oxidative stress response and thermotolerance assays. This dataset also includes statistical analysis of poly Q foci formation and paralysis.
(XLS)

File S1 Representative images for *mml-1::GFP* animals (Types I-V).
(PDF)
