# Supplementary figures and images for: The Caenorhabditis elegans Myc-Mondo/Mad Complexes Integrate Diverse Longevity Signals
Source: PLoS Genet. 2014 Apr 3;10(4):e1004278. doi: 10.1371/journal.pgen.1004278 (PMC3974684; doi:10.1371/journal.pgen.1004278)

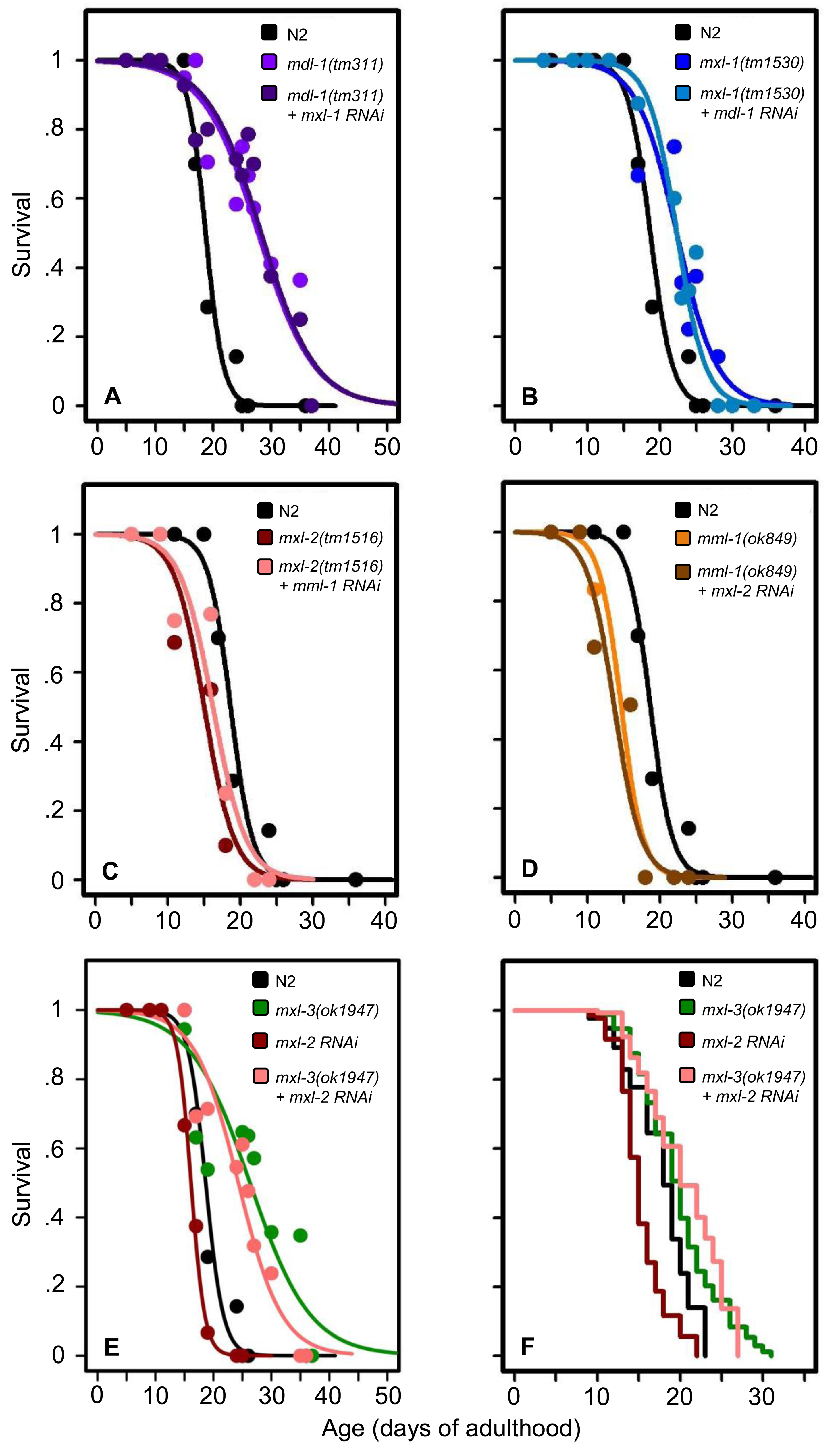

Supplement: Figure S1 — Myc-Mondo/Mad complexes likely function as heterodimers to influence longevity. (A) RNAi inactivation of mxl-1 fails to further extend the lifespan of mdl-1(tm311) mutants. (B) RNAi inactivation of mdl-1 fails to further extend the lifespan of mxl-1(tm1530) mutants. (C) RNAi inactivation of mml-1 fails to further shorten the lifespan of mxl-2(tm1516) mutants. (D) RNAi inactivation of mxl-2 fails to further shorten the lifespan of mml-1(ok849) mutants. The lack of an RNAi effect in these mutant backgrounds is likely not due to inefficacy on the part of the RNAi as the same RNAis (same bacterial culture) significantly influenced the lifespan of N2 animals within the same experiment. Furthermore, all four mutant strains responded to other RNAi inactivations within the same experiment. For details please refer to Dataset S1. (E and F) RNAi inactivation of mxl-2 fails to suppress the longevity of long-lived mxl-3(ok1947) mutants (pink versus green traces). Similar results were obtained for mml-1 RNAi (Dataset S1). The experiments in E and F were conducted by the replica set and traditional method, respectively. (TIF) [file pgen.1004278.s001.tif]

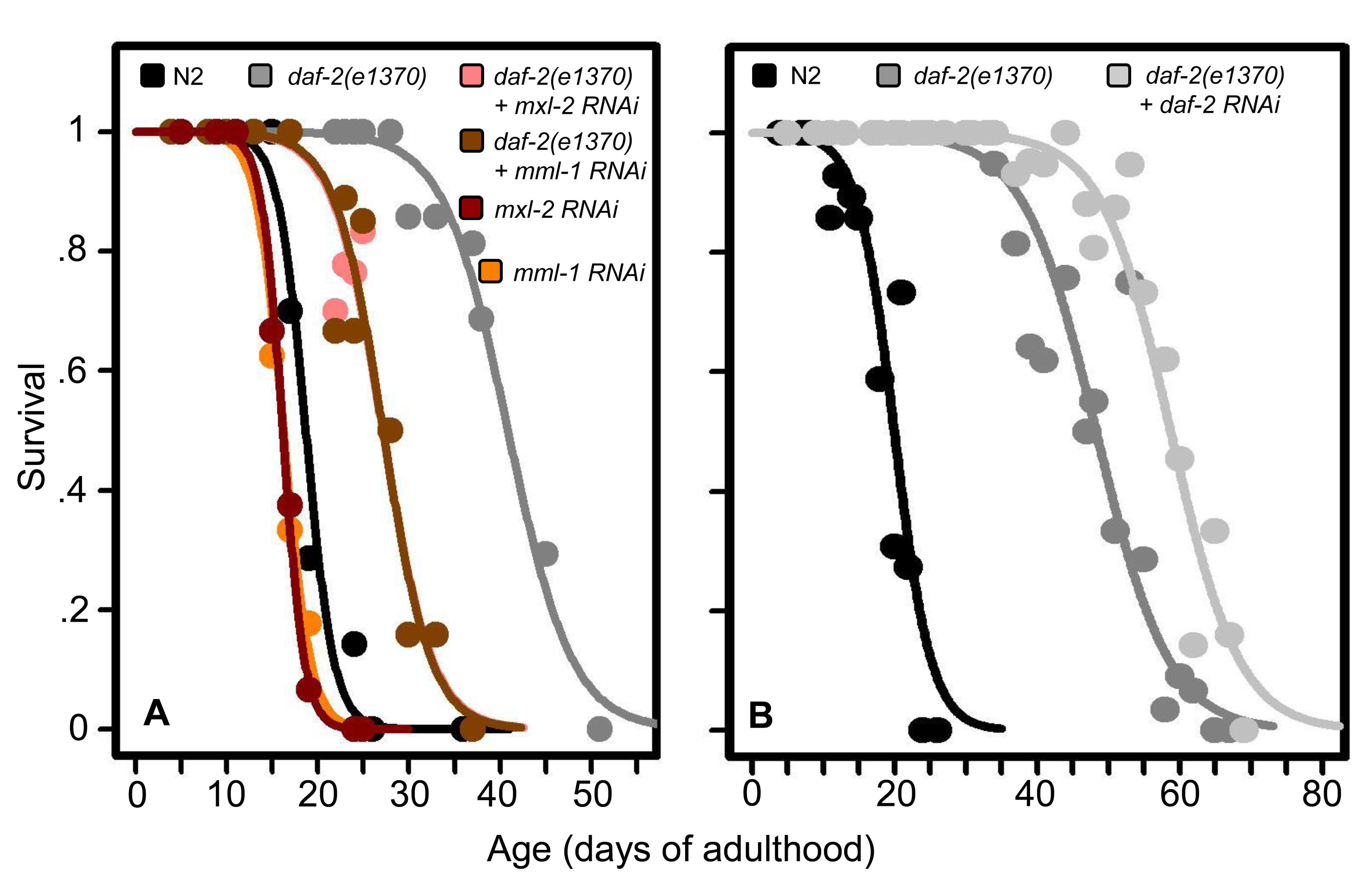

Supplement: Figure S2 — RNAi inactivation of mxl-2 and mml-1 shorten, and daf-2 RNAi extends, daf-2(e1370) mutant lifespan. (A) RNAi inactivation of mxl-2 and mml-1 significantly shorten lifespan to an extent that is similar to what is observed in daf-2(e1370);mxl-2(tm1516) mutant animals. This result corroborates the results in Figure 2B. (B) RNAi inactivation of daf-2 in daf-2(e1370) mutant significantly extends longevity. (TIF) [file pgen.1004278.s002.tif]

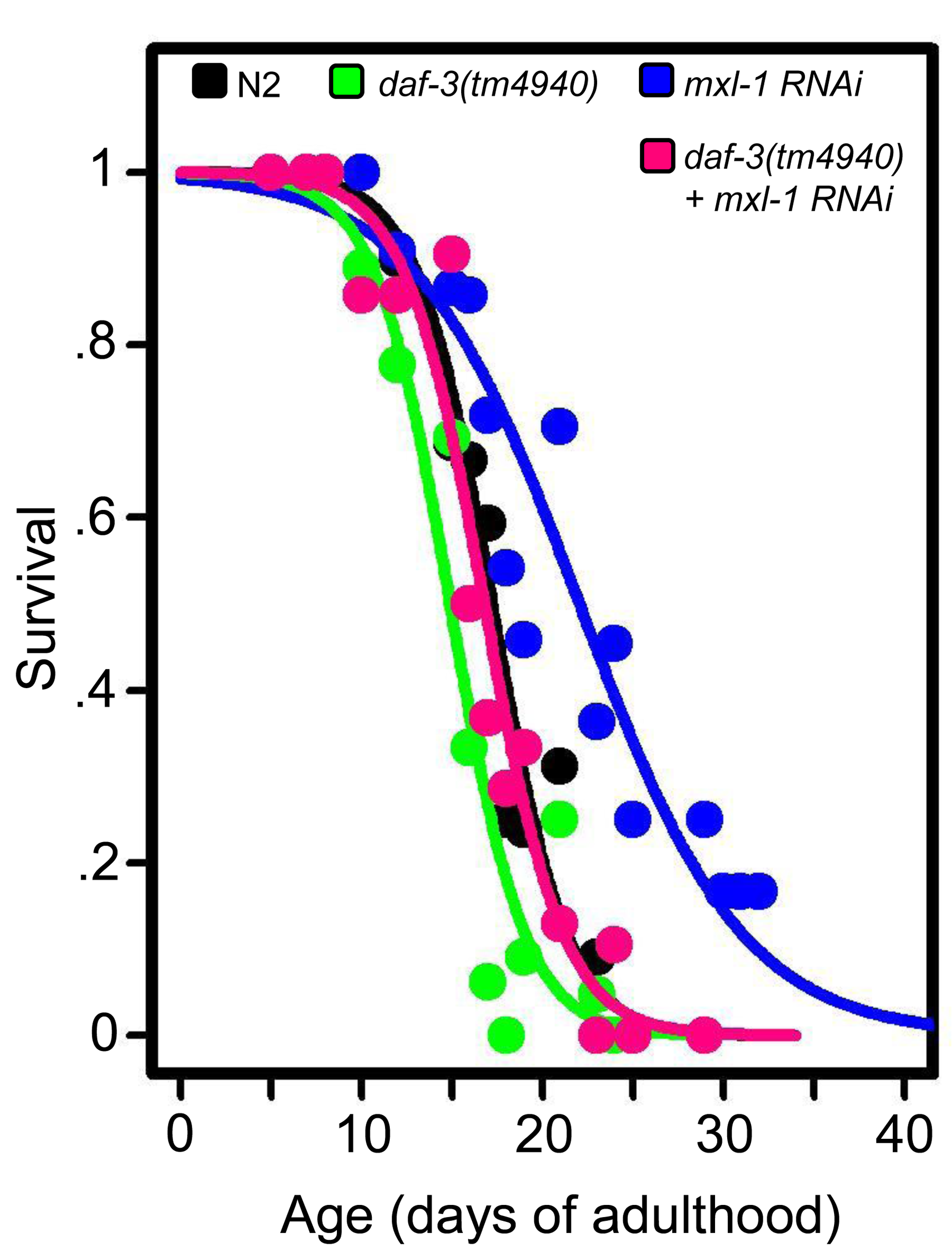

Supplement: Figure S3 — Loss of mxl-1 fails to extend longevity in the absence of daf-3 RNAi inactivation of mxl-1 robustly extends the lifespan of N2 animals but has no effect on the daf-3(tm4940) mutant. (TIF) [file pgen.1004278.s003.tif]

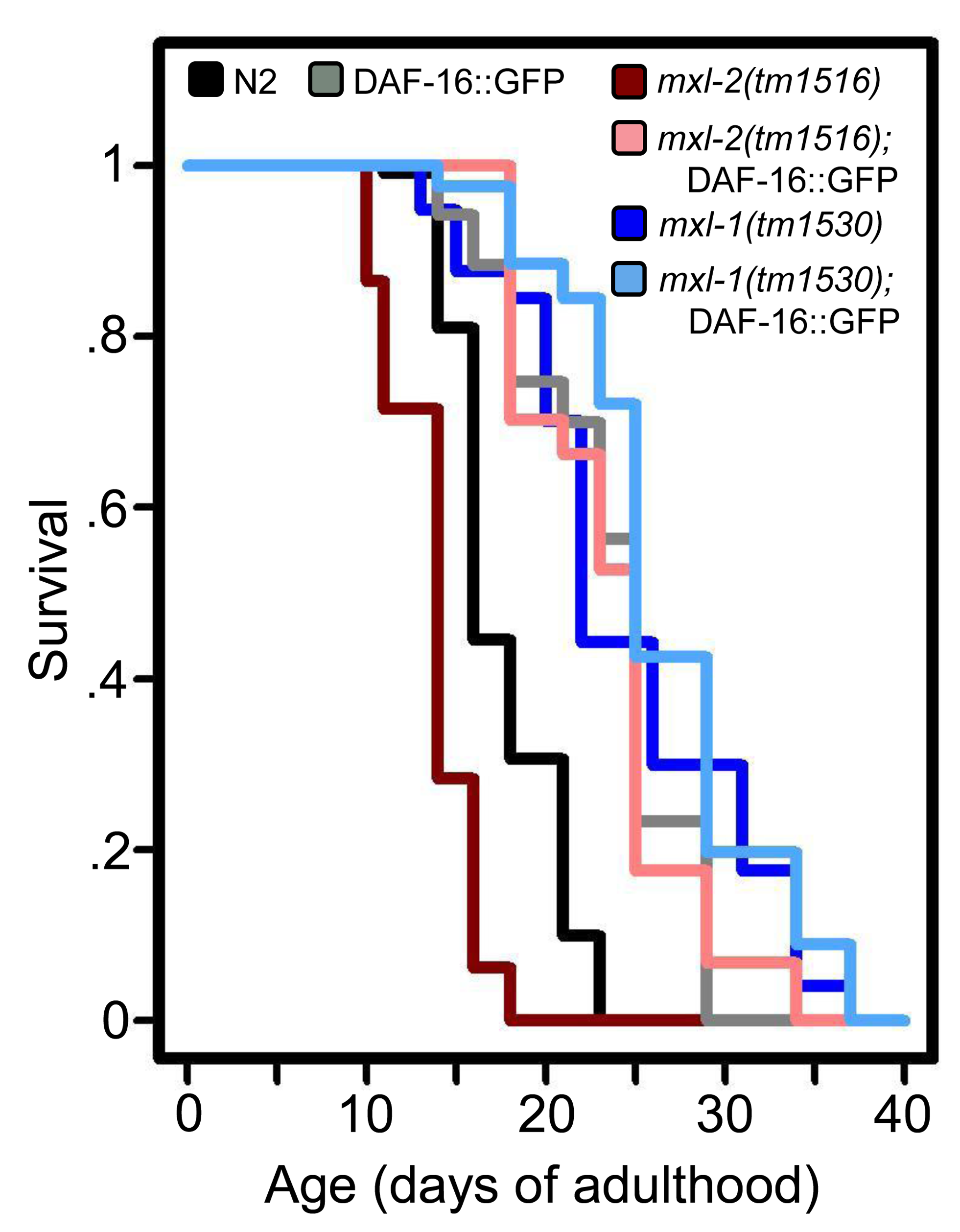

Supplement: Figure S4 — Loss of mxl-2 fails to shorten longevity in a strain that over-expresses daf-16 Lifespan was analyzed in animals expressing DAF-16::GFP translational fusion protein in the presence or absence of mxl-2 or mxl-1. Loss of mxl-2 significantly shortens the lifespan of wild-type animals (compare black and red lines); however, the lifespan of DAF-16::GFP expressing animals was identical in wild-type, mxl-2(tm1516), and mxl-1(tm1530) mutant backgrounds (compare grey, pink, and blue lines). This suggests that increased daf-16 expression can compensate for the loss of the MXL-2:MML-1 complex and that loss of the MXL-1:MDL-1 complex is not required for increased longevity when high levels of DAF-16::GFP are present. (TIF) [file pgen.1004278.s004.tif]

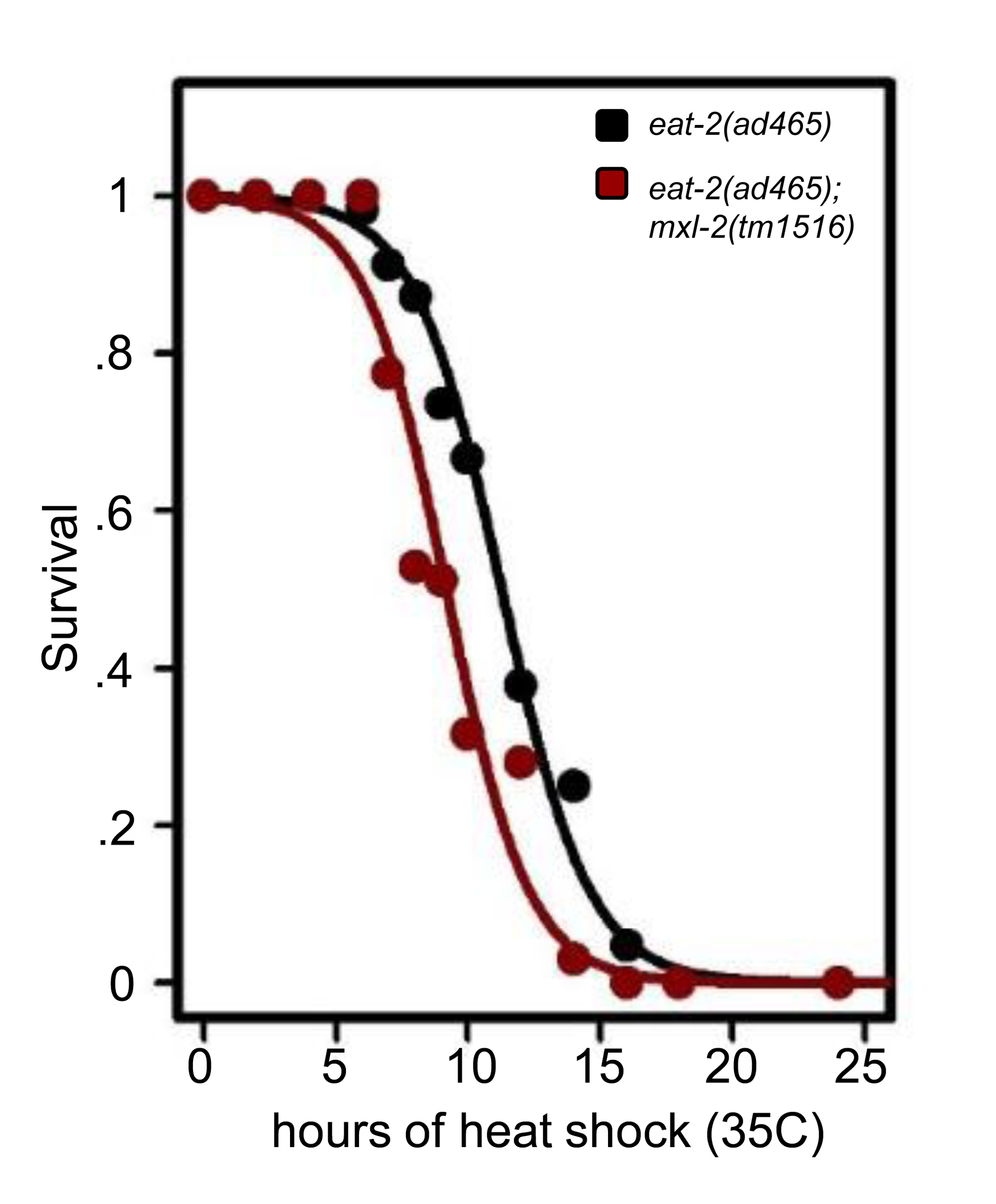

Supplement: Figure S5 — Loss of mxl-2 suppresses thermotolerance in eat-2(ad465) mutants similar to what was observed in wild-type and daf-2(e1370) mutant backgrounds loss of mxl-2 significantly weakens eat-2 mutant animals’ ability to survive thermal stress. (TIF) [file pgen.1004278.s005.tif]

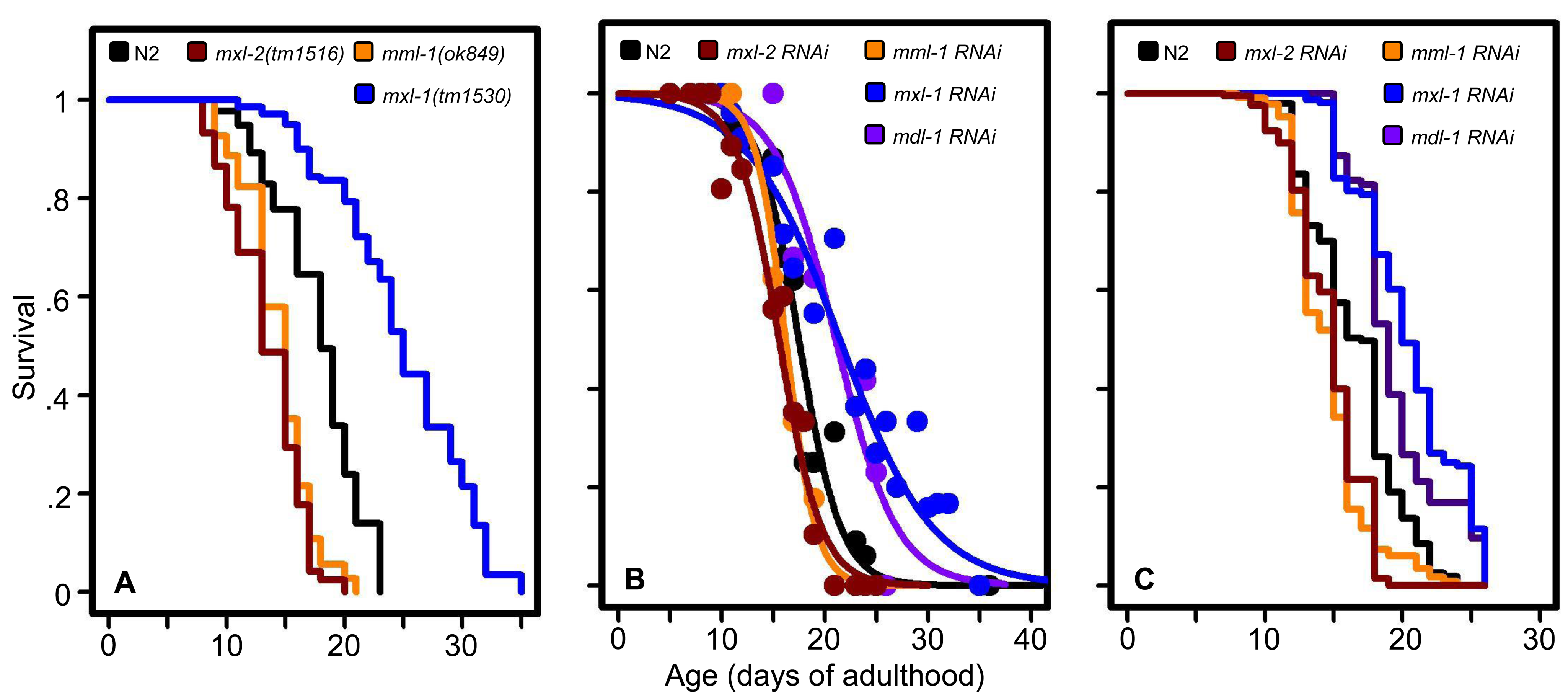

Supplement: Figure S6 — Replica set and traditional methods for scoring lifespan produce similar results. (A) Traditional lifespan analysis of mxl-2(tm1516), mml-1(ok849), and mxl-1(tm1530) mutants (compare to Figures 1B and 1C). (B and C) Replica set and traditional lifespan analysis using RNAi against all four Myc-Mondo/Mad transcription factors confirm the efficacy of RNAi clones and both methods produce comparable results. The replicate set method surveys many independent observations of whether a worm is alive or dead, then derives the median longevity (i.e. each worm is assessed whether it is alive or dead once). In contrast, the traditional lifespan method directly measures the mean lifespan of a single population tracked longitudinally in time. Both methods give a similar read for median lifespan. (TIF) [file pgen.1004278.s006.tif]

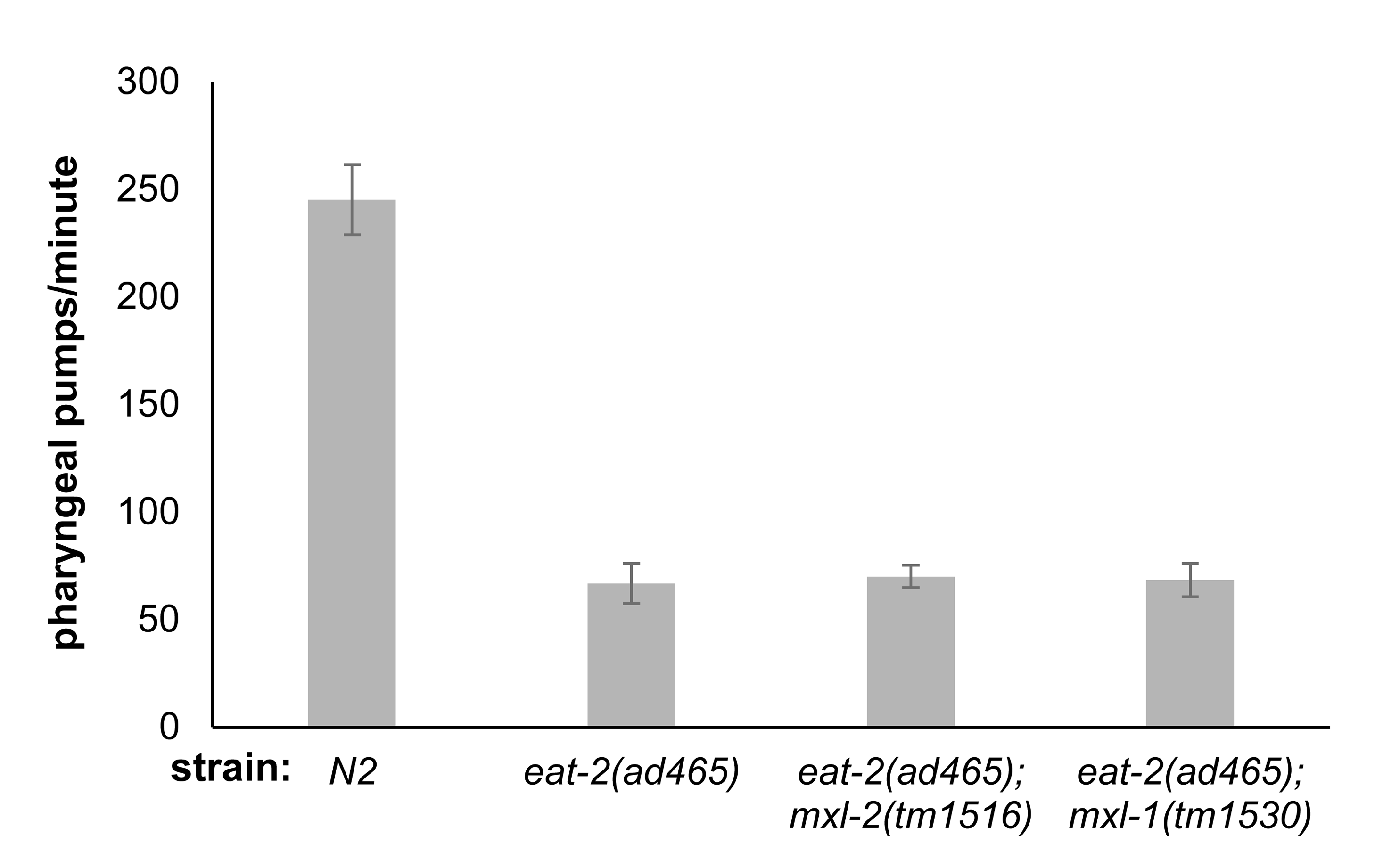

Supplement: Figure S7 — Pharyngeal pumping rates in eat-2(ad465) single and double mutants pharyngeal pumping rates in eat-2(ad465) mutants are significantly lower compared to N2 animals as previously described [26], [27]. Subsequent mutations in mxl-2 and mxl-1 do not alter pumping rates of eat-2(ad465) mutants. (TIF) [file pgen.1004278.s007.tif]
