# Supplementary material for: The Caenorhabditis elegans Myc-Mondo/Mad Complexes Integrate Diverse Longevity Signals
Source: PLoS Genet. 2014 Apr 3;10(4):e1004278. doi: 10.1371/journal.pgen.1004278 (PMC3974684; doi:10.1371/journal.pgen.1004278)
Supplement: File S1 — Representative images for mml-1::GFP animals (Types I-V). (PDF) [file pgen.1004278.s011.pdf]

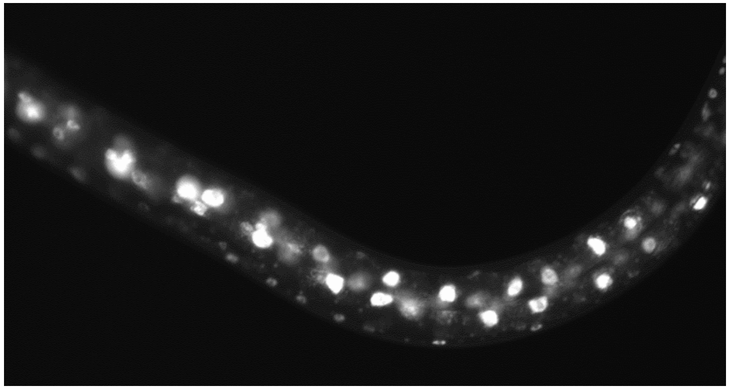
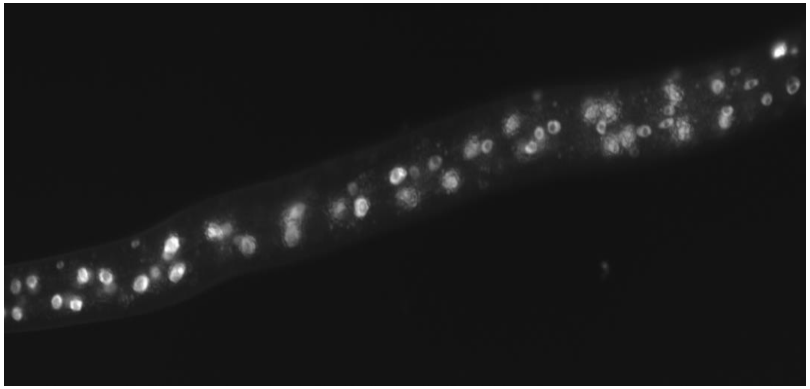
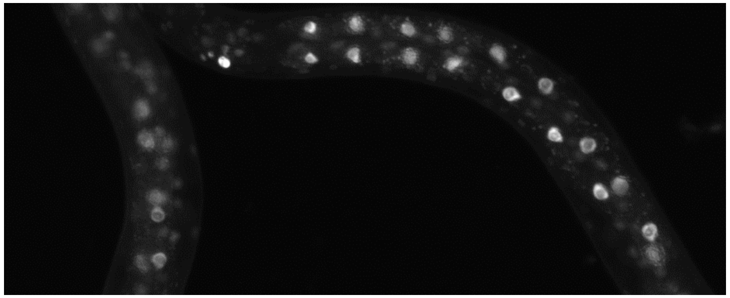
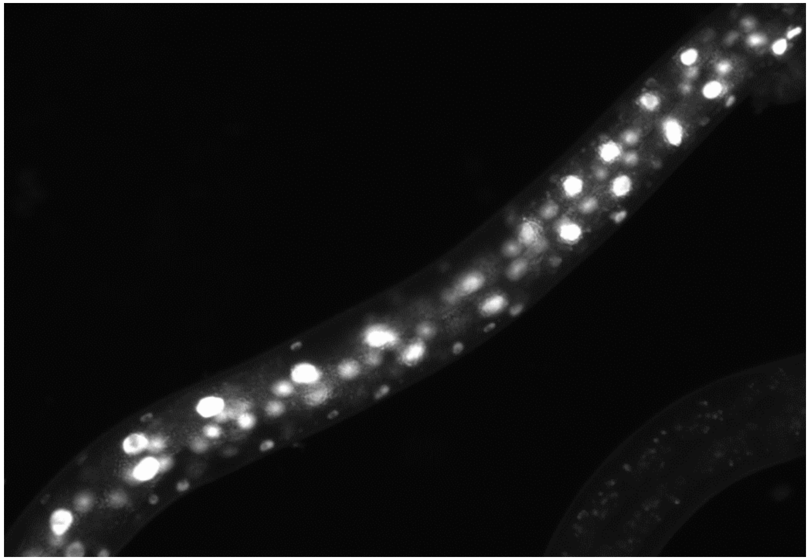

Type I: Completely nuclear

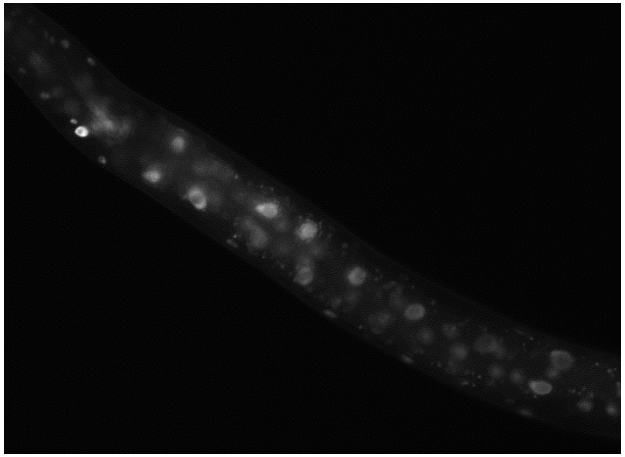

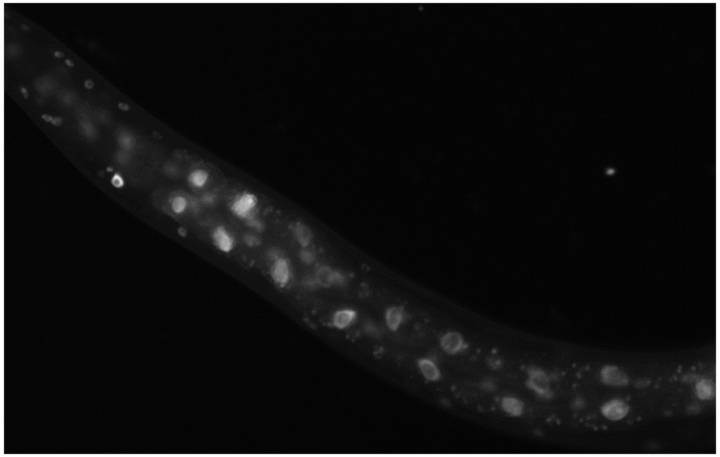

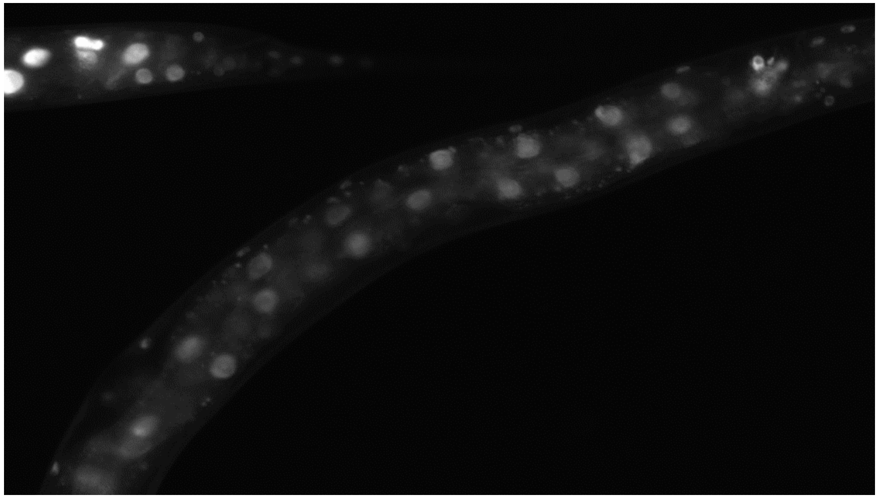

Type II: Mostly nuclear, some cytosolic

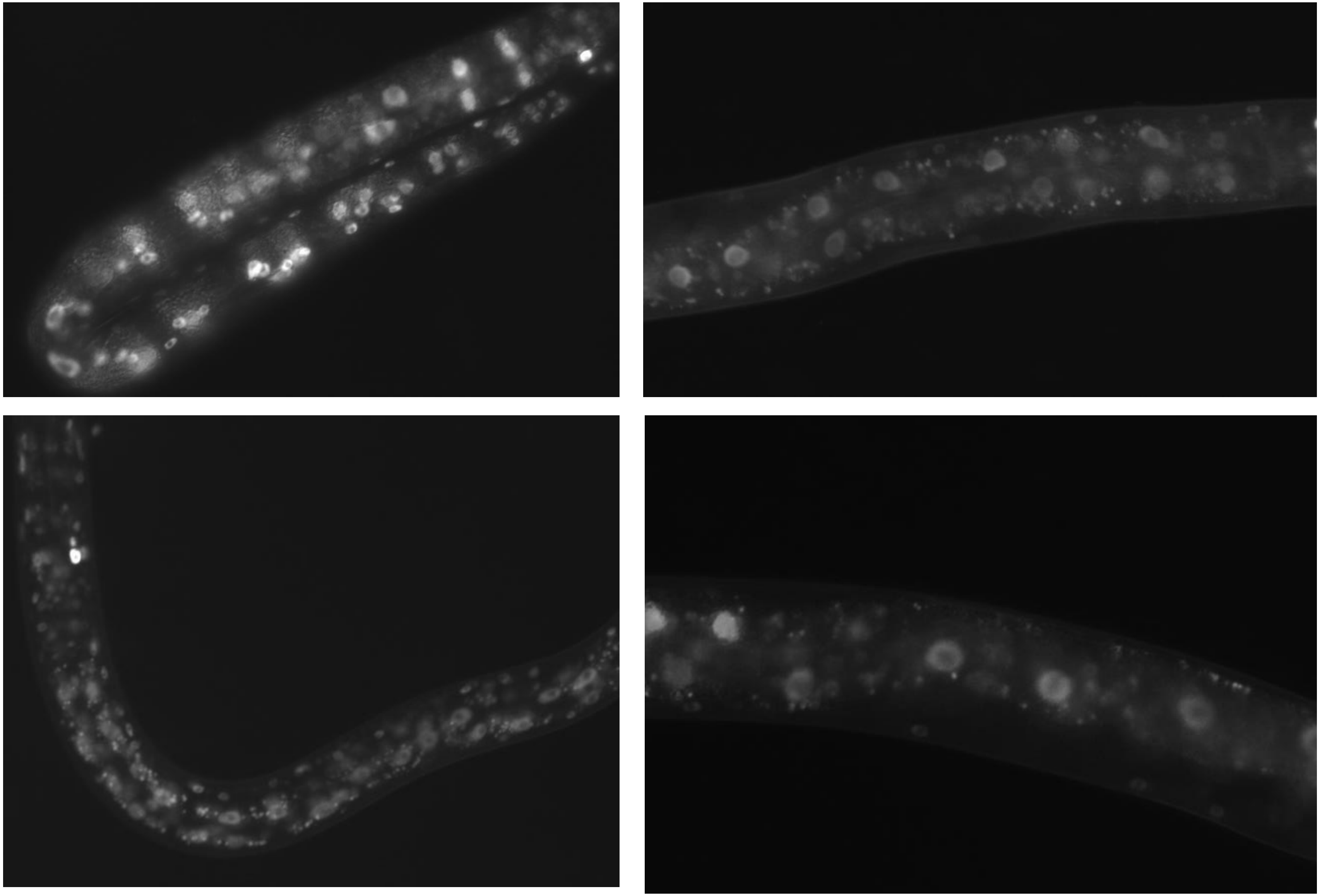

Type III: equal nuclear and cytosolic expression

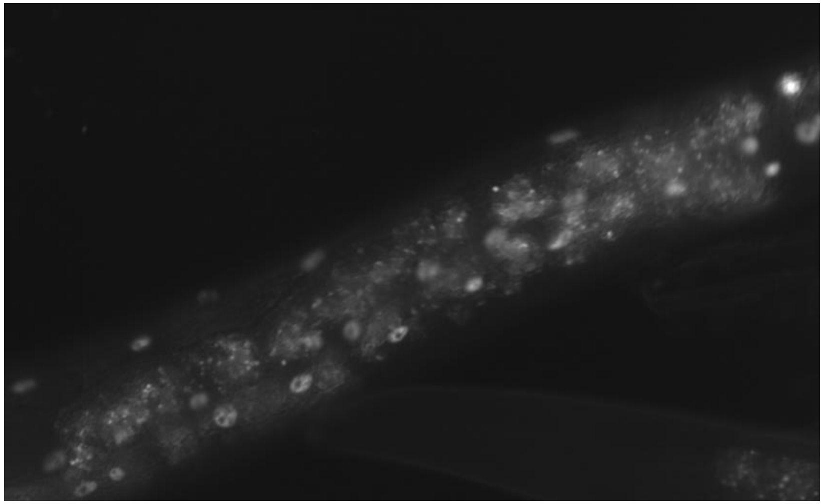

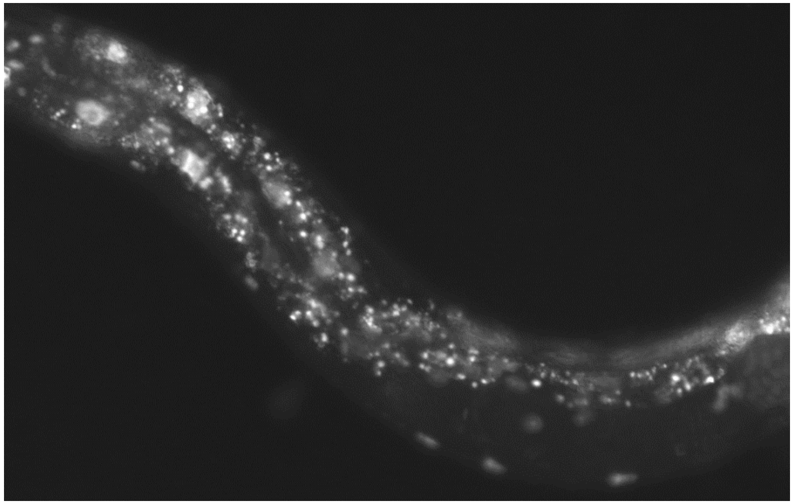

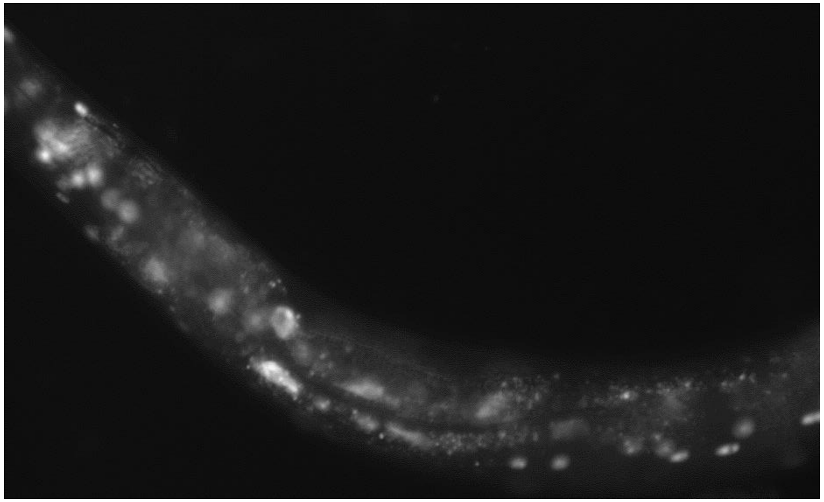

Type IV: mostly cytosolic, some nuclear

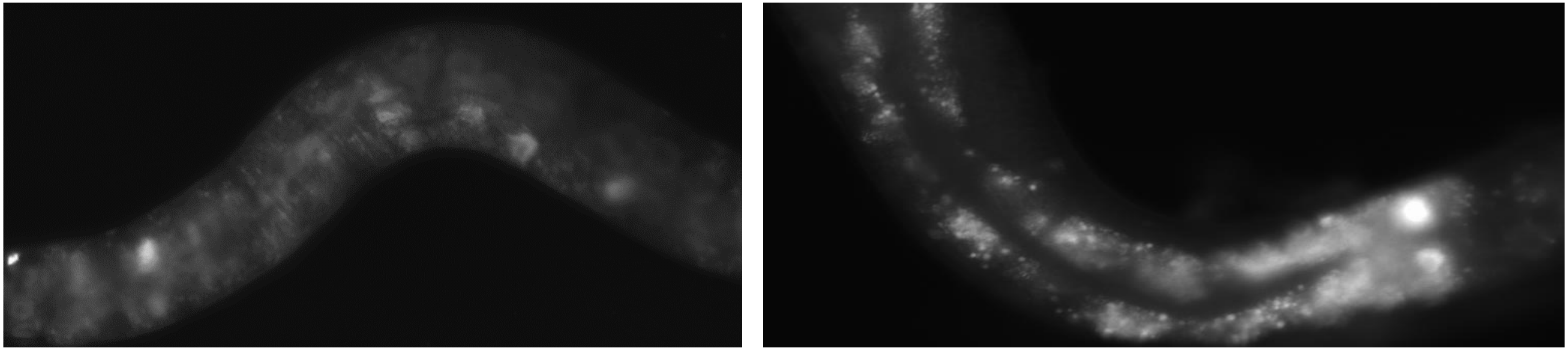

Type V: Completely cytosolic
